# Young adulthood and adulthood adiposity in relation to incidence of pancreatic cancer: a prospective study of 0.5 million Chinese adults and a meta-analysis

**DOI:** 10.1136/jech-2017-208895

**Published:** 2017-09-12

**Authors:** Yuanjie Pang, Michael V Holmes, Christiana Kartsonaki, Yu Guo, Ling Yang, Zheng Bian, Yiping Chen, Fiona Bragg, Andri Iona, Iona Y Millwood, Junshi Chen, Liming Li, Zhengming Chen

**Affiliations:** 1 Clinical Trial Service Unit and Epidemiological Studies Unit (CTSU), Nuffield Department of Population Health, University of Oxford, Oxford, UK; 2 Medical Research Council Population Health Research Unit (MRC PHRU), University of Oxford, Oxford, UK; 3 National Institute for Health Research, Oxford Biomedical Research Centre, Oxford University Hospital, Oxford, UK; 4 China National Coordinating Centre China Kadoorie Biobank, Chinese Academy of Medical Sciences, Beijing, China; 5 The China National Center for Food Safety Risk Assessment, Beijing, China; 6 School of Public Health, Peking University, Beijing, China

**Keywords:** adiposity, adulthood, young adulthood, pancreatic cancer, Chinese, meta-analysis

## Abstract

**Background:**

Adult adiposity is positively associated with pancreatic cancer in Western populations. Little is known, however, about the association in China where many have lower body mass index (BMI) or about the relevance of young adulthood adiposity for pancreatic cancer in both Western and East Asian populations.

**Methods:**

The China Kadoorie Biobank (CKB) recruited 512 891 adults aged 30–79 years during 2004–2008, recording 595 incident cases of pancreatic cancer during 8-year follow-up. Cox regression yielded adjusted HRs for pancreatic cancer associated with self-reported young adulthood (mean ~25 years) BMI and with measured adulthood (mean ~52 years) BMI and other adiposity measures (eg, waist circumference (WC)). These were further meta-analysed with published prospective studies.

**Results:**

Overall, the mean BMI (SD) was 21.9 (2.6) at age 25 years and 23.7 (3.3) kg/m^2^ at age 52 years. Young adulthood BMI was strongly positively associated with pancreatic cancer in CKB (adjusted HR=1.36, 95% CI 1.16 to 1.61, per 5 kg/m^2^ higher BMI) and in meta-analysis of CKB and four other studies (1.18, 1.12 to 1.24). In CKB, there was also a positive association of pancreatic cancer with adulthood BMI (1.11, 0.97 to 1.27, per 5 kg/m^2^), similar in magnitude to that in meta-analyses of East Asian studies using measured BMI (n=2; 1.08, 0.99 to 1.19) and of Western studies (n=25; 1.10, 1.06 to 1.12). Likewise, meta-analysis of four studies, including CKB, showed a positive association of adulthood WC with pancreatic cancer (1.10, 1.06 to 1.14, per 10 cm).

**Conclusions:**

In both East Asian and Western populations, adiposity was positively associated with risk of pancreatic cancer, with a somewhat stronger association for young than late-life adiposity.

## Introduction

Pancreatic cancer is the sixth leading cause of cancer-related death globally,^1 2^ with an extremely poor survival rate.^2 3^ In recent decades, the mean levels of adiposity, usually measured by body mass index (BMI), have been increasing steadily in China and many other East Asia countries.^4^ This has been accompanied by an increase in incidence rates of pancreatic cancer (and diabetes and several other diseases) in adult populations.^5–7^ In 2012, the World Cancer Research Fund concluded that both adult general (eg, BMI) and abdominal adiposity (eg, waist circumference (WC)) are associated with increased risks of pancreatic cancer.^8^ Adiposity is also linked to other risk factors for pancreatic cancer, such as insulin resistance and diabetes.^9^

Previous studies of adiposity and pancreatic cancer mainly involved Western populations and used mostly measured or self-reported BMI, at middle age or old age.^10 11^ Questions remain, however, about the associations of pancreatic cancer with measures of central adiposity (eg, WC and waist and hip ratio (WHR)) and with young adulthood BMI, usually defined as BMI at age 18–25 years. Compared with adulthood BMI, young adulthood BMI is less affected by reverse causality (ie, weight loss induced by preclinical pancreatic cancer) and may also capture early life exposures in a similar way as adult attained height.^8^ In China and other East Asian populations, the mean levels of adiposity are still much lower than in Western populations, but a higher proportion tend to have central, rather than general, obesity compared with their counterparts in the West.^4 12^ Reliable prospective evidence is, therefore, needed to assess whether adiposity is associated differently with pancreatic cancer in Chinese and other East Asian populations compared with Western populations.

We report findings among 512 891 adults in the prospective China Kadoorie Biobank (CKB) study. The main objectives of the study were to investigate the associations of (A) young adulthood BMI and (B) adulthood general (BMI, fat percentage and height-adjusted weight) and central (WC and WHR) adiposity with incident pancreatic cancer. To help compare and quantify reliably the strength of the association across different populations, we also meta-analysed the present study with published prospective studies on adiposity and pancreatic cancer.

## Methods

### Study population

Details of the CKB design, survey methods and population characteristics have been described elsewhere.^13^ Briefly, 512 891 participants (210 222 men and 302 669 women) aged 30–79 years were recruited into the study from 10 localities (five urban and five rural) in China during 2004–2008. The study areas were selected to provide diversity in risk exposure and disease patterns, while taking into account population stability, quality of mortality and morbidity registries, capacity and long-term commitment within the areas. All participants provided written informed consent. Prior international, national and regional ethical approvals were obtained.

### Data collection

At local study assessment clinics, participants completed an interviewer-administered laptop-based questionnaire on sociodemographic characteristics, smoking, alcohol consumption, diet, physical activity, personal and family medical history and current medication. A range of physical measurements were recorded by trained technicians, including height, weight, hip and waist circumference, bioimpedance, lung function, blood pressure and heart rate, using calibrated instruments with standard protocols.

All measurements were made once by trained technicians while participants were wearing light clothes and no shoes. Standing height was measured to the nearest 0.1 cm using a stadiometer. Weight was measured to the nearest 0.1 kg using a body composition analyser (TANITA-TBF-300GS; Tanita), with subtraction of weight of clothing by 0.5 kg in summer, 1.0 kg in spring/autumn and 2.0–2.5 kg in winter. WC and hip circumference (HC) were measured to the nearest 0.1 cm using a soft, non-stretchable tape. HC was measured at the maximum circumference around the buttocks. WHR was the ratio of WC to HC. Body fat percentage was the fraction of total weight that was estimated to be fat weight by the Tanita body composition analyser using proprietary algorithms.

Adulthood BMI was calculated as the measured weight in kilograms divided by the square of the measured height in metres. Young adulthood BMI was calculated using the recalled weight at age 25 years and the measured height at baseline.

### Follow-up for mortality and morbidity

The vital status of each participant was determined periodically through China Centre for Disease Control and Prevention’s (CDC) Disease Surveillance Points system,^14^ supplemented by regular checks against local residential records and health insurance records and by annual active confirmation through street committees or village administrators. In addition, information about major diseases and any episodes of hospitalisation was obtained through linkages, via each participant’s unique national identification number, with disease registries (for cancer, ischaemic heart disease, stroke and diabetes) and national health insurance claims databases. All events were coded using International Classification of Diseases, 10th Revision (ICD-10) by trained staff who were blinded to baseline information.^13^ By 1 January 2015, 30 582 (6%) participants had died, 3898 (0.8%) were lost to follow-up and 21 266 (4.2%) had developed cancer, including 598 (0.12%) with pancreatic cancer (ICD-10 C25).

### Statistical analysis

The present study excluded participants with a history of cancer at baseline (n=2577). For analyses of adulthood adiposity, we further excluded pancreatic cancer cases (n=101) that occurred during the first 2 years of follow-up to minimise possible effects of weight loss due to undiagnosed pancreatic cancer. The prevalence and mean values of baseline characteristics were calculated according to young adulthood and adulthood BMI categories at baseline, using direct standardisation to the age (in 5-year age groups), sex and study area structure of the CKB population. Incidence rates of pancreatic cancer in all participants were calculated with direct standardisation by sex and region to the CKB study population.

Cox regression models were used to obtain adjusted HRs of pancreatic cancer associated with adiposity, stratified by age at risk (5 year age groups), sex and study area (10 areas) and adjusted for education (four groups: no formal school, primary school, middle/high school or college/university), smoking (three groups: never, occasional or ever regular) and alcohol (five groups: abstainers, ex-weekly drinkers, reduced-intake drinkers, occasional drinkers or weekly drinkers). For the categorical analyses, young adulthood and adulthood BMI were modelled using cut points based on their corresponding distributions (<20.0, 20.0–<22.5, 22.5–<25.0, 25.0–<27.0 and ≥27.0 kg/m^2^). BMI was also modelled as a continuous variable to estimate effects per 5 kg/m^2^ or per one SD higher value. For height-adjusted weight, standing height was also included in the model as a continuous variable. WC, WHR, body fat percentage, height-adjusted weight, height and leg length were modelled as overall tertiles and per one SD increase. HRs for each anthropometric category are presented along with ‘floating’ standard errors, so that each HR has a 95% CI that appropriately reflects the number of subjects and pancreatic cancer cases in that category.^15^

Statistical analyses were conducted using SAS V.9.3, R V.2.14.2 and Stata V.13.0.

### Meta-analysis of published studies

We followed Preferred Reporting Items for Systematic Review and Meta-Analysis guidelines for conducting a systematic review and meta-analysis.^16^ PubMed and Embase were searched for studies published in English from database inception to September 2016. The precise search terms are provided in the online supplementary data. Inclusion criteria were prospective cohort studies, case–cohort studies or nested case–control studies reporting the association between adiposity and pancreatic cancer incidence or mortality. Bibliographies of included studies and related reviews were manually searched for additional eligible articles.

10.1136/jech-2017-208895.supp1 Supplementary file 1

Our systematic review identified 1489 eligible studies, of which 31 met our inclusion criteria (online supplementary figure 1). These included 31 (30 prospective, 1 nested case–control) studies reporting adulthood BMI, four (two prospective, one nested case–control and one US-based meta-analysis) studies reporting young adulthood BMI and three reporting WC and WHR. Characteristics of the included studies are provided in online supplementary tables 2 and 3.

Of the 31 studies included for adulthood BMI (online supplementary data), 9 used measured BMI and 20 used self-reported BMI. Twelve studies were from North America, 13 from Europe and 6 from Asia, and in total they included 19 680 pancreatic cancer cases. Two studies used the same population with different age restrictions (age range 45–95 years^17^ vs 30–95 years^18^), and we included the one by Berrington de González *et al*^18^ as the mean age was more comparable with the majority of included studies.

Of the four studies included for meta-analysis of young adulthood BMI, all used self-reported BMI, as in the present study, and there were a total of 4123 pancreatic cancer cases (online supplementary data).

If available, we used the originally reported relative risks (RRs) per 5 kg/m^2^ higher BMI or estimated them by quantifying the study-specific linear trends between exposure and outcome using the method described by Greenland and Longnecker^19^ (which allows for non-independence of RR estimates within each study). We then pooled the linear trends using fixed effect meta-analysis. As a sensitivity analysis, we also used a random effects model (online supplementary table 1). The main subgroup analyses included investigation by geographical region, sex, BMI assessment method (measured or self-reported), different adjustment in the statistical models, whether studies excluded initial years of follow-up, mean BMI, mean age and median follow-up. Meta-regression analyses were conducted to assess heterogeneity between subgroups. Heterogeneity between studies was assessed by *I*^2^ and Cochran’s *Q* test. Publication bias was assessed visually by inspecting the funnel plots for asymmetry and tested with Egger’s test,^20^ with the result considered to indicate small study effects when *p*<0.05.

Apart from BMI, three papers reported the RR per 10 cm higher WC and the RR per 0.1 unit higher WHR. We pooled the estimated RRs for WC and WHR separately using fixed effect meta-analysis.

## Results

Among the 510 314 participants, the mean (SD) baseline age was 51.5 (10.7) years, and 59% were women. The mean (SD) measured BMI at baseline (ie, adulthood BMI) and BMI at age 25 years (ie, young adulthood BMI) was 23.7 (3.3) kg/m^2^ and 21.9 (2.6) kg/m^2^, respectively. Among men, mean adulthood BMI decreased with increasing age, whereas among women, it increased until about 50–59 years then fell afterwards (online supplementary figure 2). In both sexes, young adulthood BMI was higher among those who were older at baseline. Participants with higher adulthood or young adulthood BMI were more likely to have higher systolic blood pressure and blood glucose and to have prevalent diabetes and a family history of diabetes (table 1).

**Table 1:** Participant characteristics by adulthood BMI and young adulthood BMI in CKB

Variable	Adulthood BMI (age 52 years), kg/m^2^				Young adulthood BMI (age 25 years), kg/m^2^			
	<20.0 (n=66 595)	20.0–23.4 (n=192 058)	23.5–26.9 (n=171 162)	≥27.0 (n=83 074)	<20.0 (n=98 288)	20.0–23.4 (n=226 932)	23.5–26.9 (n=89 941)	≥27.0 (n=15 315)
Adulthood BMI (SD), kg/m^2^	18.7 (1.1)	21.8 (0.9)	25.0 (0.9)	29.3 (2.0)	22.45 (3.1)	23.6 (3.1)	24.9 (3.4)	26.7 (3.9)
Young adulthood BMI (SD), kg/m^2^	20.5 (2.3)	21.5 (2.3)	22.2 (2.5)	23.2 (2.8)	18.8 (1.1)	21.7 (1.0)	24.8 (0.9)	28.6 (1.7)
Age and lifestyle factors								
Age (SD), year	52.5 (11.8)	50.8 (10.8)	51.3 (10.4)	51.7 (10.2)	49.4 (10.1)	50.3 (10.2)	52.8 (10.4)	55.3 (10.2)
Female (%)	55.1	57.9	59.5	63.6	63.7	52.5	60.0	63.9
Urban region (%)	31.0	38.8	49.1	56.0	53.8	47.2	45.1	47.9
≥9 years of education (%)	48.5	49.7	50.2	48.7	54.1	51.4	47.4	43.8
Household income ≥35 000 yuan/year (%)	14.8	17.2	19.3	20.1	20.2	18.7	17.2	16.7
Ever regular smoking (%)								
Male	76.0	70.1	63.7	61.4	57.6	59.0	59.8	60.7
Female	4.2	2.9	2.5	2.6	1.8	1.6	1.9	2.6
Weekly drinking (%)								
Male	31.5	34.0	33.1	32.4	32.9	33.7	34.3	32.4
Female	2.1	2.1	2.1	1.9	2.3	2.1	2.1	2.3
Blood pressure and anthropometry								
SBP (SD), mm Hg	122.9 (20.9)	128.1 (20.4)	133.5 (20.8)	139.4 (21.3)	129.6 (20.5)	130.3 (20.4)	133.3 (21.3)	138.0 (22.6)
RPG (SD), mmol/L	5.9 (2.1)	5.9 (2.1)	6.1 (2.4)	6.4 (2.6)	6.0 (2.0)	6.0 (2.1)	6.2 (2.6)	6.9 (2.7)
Total physical activity (SD), MET hour/day	21.4 (13.9)	21.6 (14.2)	20.8 (13.8)	19.8 (13.0)	20.5 (13.1)	21.1 (14.2)	21.5 (14.3)	20.8 (13.4)
Waist circumference (SD), cm	68.3 (5.2)	75.8 (5.7)	83.9 (6.1)	93.0 (7.4)	78.5 (9.6)	80.1 (9.5)	82.5 (9.7)	86.5 (10.5)
Hip circumference (SD), cm	83.6 (4.3)	88.2 (4.4)	93.0 (4.6)	99.0 (5.9)	89.8 (6.6)	90.9 (6.7)	92.4 (7.1)	94.7 (7.9)
Waist to hip ratio (SD)	0.82 (0.06)	0.86 (0.06)	0.90 (0.06)	0.94 (0.07)	0.87 (0.07)	0.88 (0.07)	0.89 (0.07)	0.91 (0.07)
Height (SD), cm	158.6 (8.3)	158.6 (8.1)	158.8 (8.3)	158.9 (8.5)	160.1 (8.4)	158.8 (8.1)	157.8 (7.7)	157.1 (9.1)
Leg length (SD), cm	73.9 (4.9)	73.6 (4.7)	73.3 (4.8)	72.9 (4.9)	74.3 (4.9)	73.5 (4.8)	72.8 (4.5)	72.2 (5.0)
Fat percentage (SD), %	18.9 (4.9)	24.9 (5.7)	30.7 (6.2)	36.6 (7.6)	26.5 (7.8)	27.9 (8.1)	29.6 (8.8)	32.1 (9.1)
Prior disease history (%)								
Self-reported diabetes	1.8	2.7	3.5	4.3	2.5	2.8	4.0	9.2
Screen-detected diabetes	1.6	2.0	3.0	4.8	2.5	2.6	3.1	5.0
Family history of diabetes	3.7	4.4	5.3	6.0	5.2	4.9	5.0	6.7
Family history of cancer	13.0	13.7	14.3	14.6	14.4	14.3	14.3	14.2

All values were based on baseline, except for young adulthood BMI. Results were adjusted for age, region and sex (where appropriate). For young adulthood BMI, regular smoker referred to smoking status at age 25 years. Young adulthood BMI was missing in 82 413 participants.

BMI, body mass index; CKB, China Kadoorie Biobank; MET, metabolic equivalent of task; RPG, random plasma glucose; SBP, systolic blood pressure.

**Figure 1:**
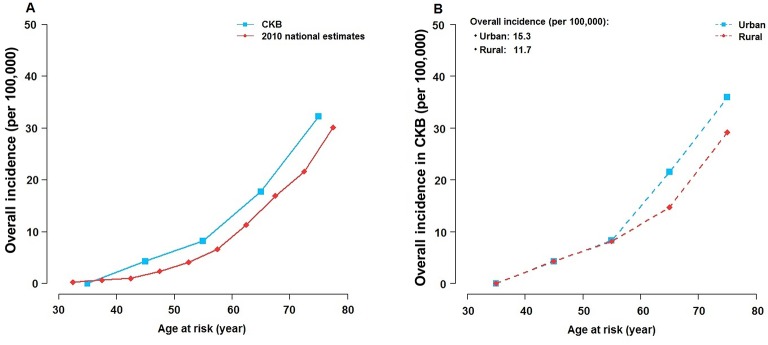
Age-specific incidence of pancreatic cancer in CKB versus Chinese national estimate in 2010. Incidence in CKB was calculated for each age at risk category and standardised by sex and region, where appropriate. Chinese national estimate was from the Global Burden of Disease Study 2010.^21^ The average incidence in CKB was 15.3 and 11.7 per 100 000 in urban and rural areas, respectively. Figure 1A shows CKB versus Chinese national estimate. Figure 1B shows urban versus rural areas in CKB. CKB, China Kadoorie Biobank.

During approximately 4.1 million person-years of follow-up, 585 participants developed pancreatic cancer between the ages of 35–79 years, and the incidence rose steeply with age. Incidence rates in CKB were comparable with the national estimate from China in 2010 (figure 1A),^21^ with age-standardised and area-standardised incidence rates 15.3 per 100 000 in urban areas and 11.7 per 100 000 in rural areas (figure 1B).

### BMI and risk of pancreatic cancer

**Figure 2:**
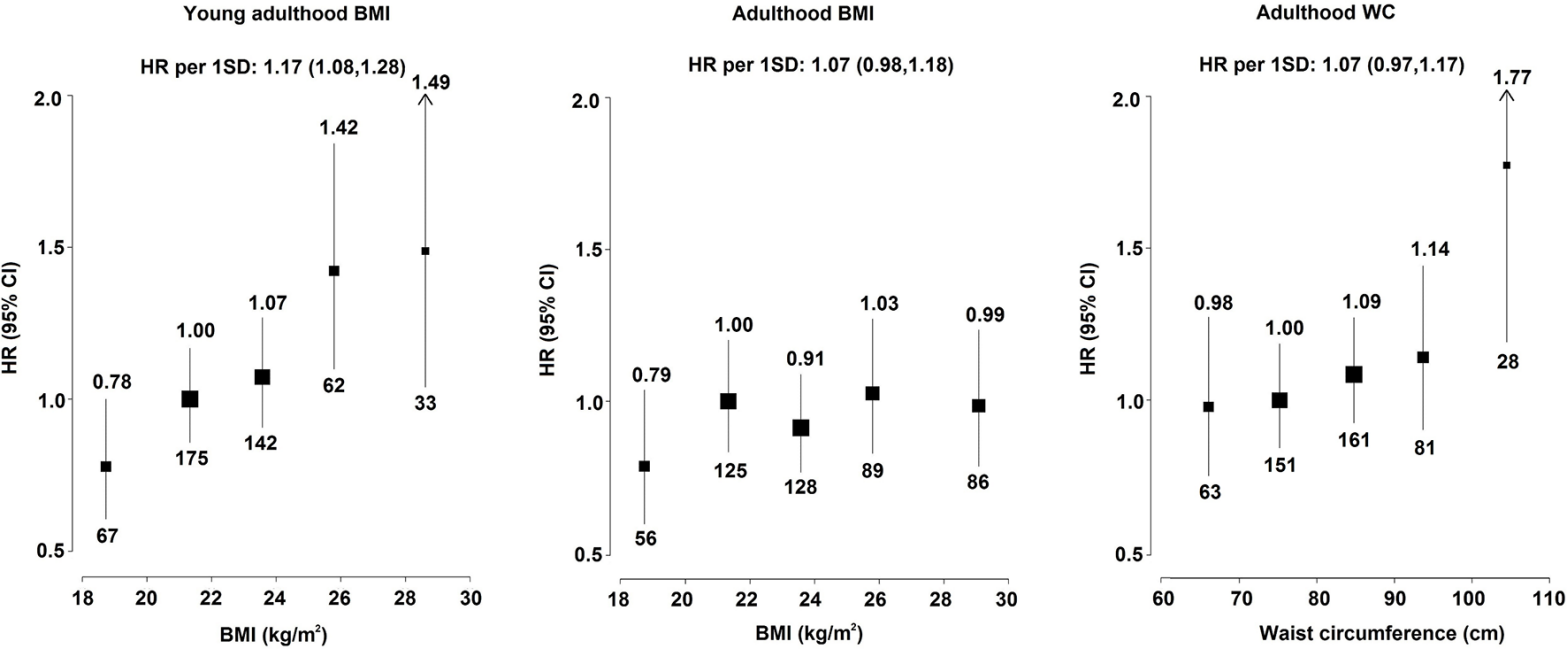
Adjusted HRs for pancreatic cancer by measures of adiposity at young adulthood (aged ~25 years) and adulthood (aged ~52 years) in CKB. Young adulthood and adulthood BMI were classified as <20.0, 20.0–22.4 (reference), 22.5–24.9, 25.0–26.9 and ≥27.0 kg/m^2^. WC was classified as <70, 70–<80, 80–<90, 90–<100 and ≥100 cm. The sizes of the boxes are proportional to the inverse of the variance of the log HRs. The models were stratified by age at risk, sex and study area, and adjusted for education, smoking and alcohol. For young adulthood BMI, smoking status at age 25 years was used in the adjustment. SD was 3.38 kg/m^2^ for adulthood BMI, 2.59 kg/m^2^ for young adulthood BMI and 9.74 cm for WC. Numerical values above the 95% CI represent the HR and values beneath the 95% CI represent the number of cases of pancreatic cancer in each group. The HRs and CIs are shown in online supplementary table 7. BMI, body mass index; CKB, China Kadoorie Biobank; WC, waist circumference.

Young adulthood BMI was strongly positively associated with risk of pancreatic cancer, after adjusting for adulthood BMI, with adjusted HRs of 0.78 (0.61 to 0.99), 1.00 (0.86 to 1.16) (reference), 1.07 (0.91 to 1.26), 1.42 (1.10 to 1.83) and 1.49 (1.05 to 2.11) for those with BMI of <20.0, 20.0–22.4, 22.5–24.9, 25.0–26.9 and ≥27.0 kg/m^2^. It was estimated that each 5 kg/m^2^ higher young adulthood BMI was associated with HR of 1.36 (95% CI 1.16 to 1.61) for pancreatic cancer late in life (figure 2 and online supplementary table 7). The association between young adulthood BMI and risk of pancreatic cancer was similar after adjusting for other adulthood adiposity measures instead of adulthood BMI, including WC, HC, WHR and body fat percentage (online supplementary table 4). Adulthood BMI showed a non-significant positive association with pancreatic cancer, with adjusted HR of 1.11 (0.97 to 1.27) per 5 kg/m^2^ higher BMI. For a one SD higher BMI, again the HR was stronger for young adulthood BMI (1.17, 1.08 to 1.28) than for adulthood BMI (1.07, 0.98 to 1.18). Additional adjustment for diabetes, a possible mediator on the pathway between adulthood adiposity and pancreatic cancer, did not alter the results (HR 1.08, 0.95 to 1.24, per 5 kg/m^2^ higher adulthood BMI), nor did adjustment for WC (HR 1.11 vs 1.09 per 5 kg/m^2^ higher adulthood BMI). As shown in online supplementary tables 5 and 6, the associations of adulthood BMI and young adulthood BMI with pancreatic cancer risk did not differ by smoking status (*p* for heterogeneity 0.43 and 0.19, respectively).

### Other measures of adiposity and risk of pancreatic cancer

There was a non-significant positive association between adulthood WC and risk of pancreatic cancer, with adjusted HR of 1.07 (0.97 to 1.17) per one SD higher WC (equivalent to 9.74 cm), similar in strength to that for adulthood BMI. Despite this, individuals with WC ≥100 cm appeared to have ~80% (HR=1.77, 1.26 to 2.50) excess risk compared with those with WC 70–80 cm (figure 2). HC, WHR, body fat percentage and height-adjusted weight measured during adulthood were not associated with risk of pancreatic cancer (online supplementary figure 3 and table 8). Both standing height and leg length showed positive trends with risk of pancreatic cancer, with HRs comparing top versus bottom tertile of 1.29 (1.04 to 1.57) and 1.16 (0.97 to 1.39), respectively (online supplementary figure 3).

### Meta-analysis of CKB with published studies

**Figure 3:**
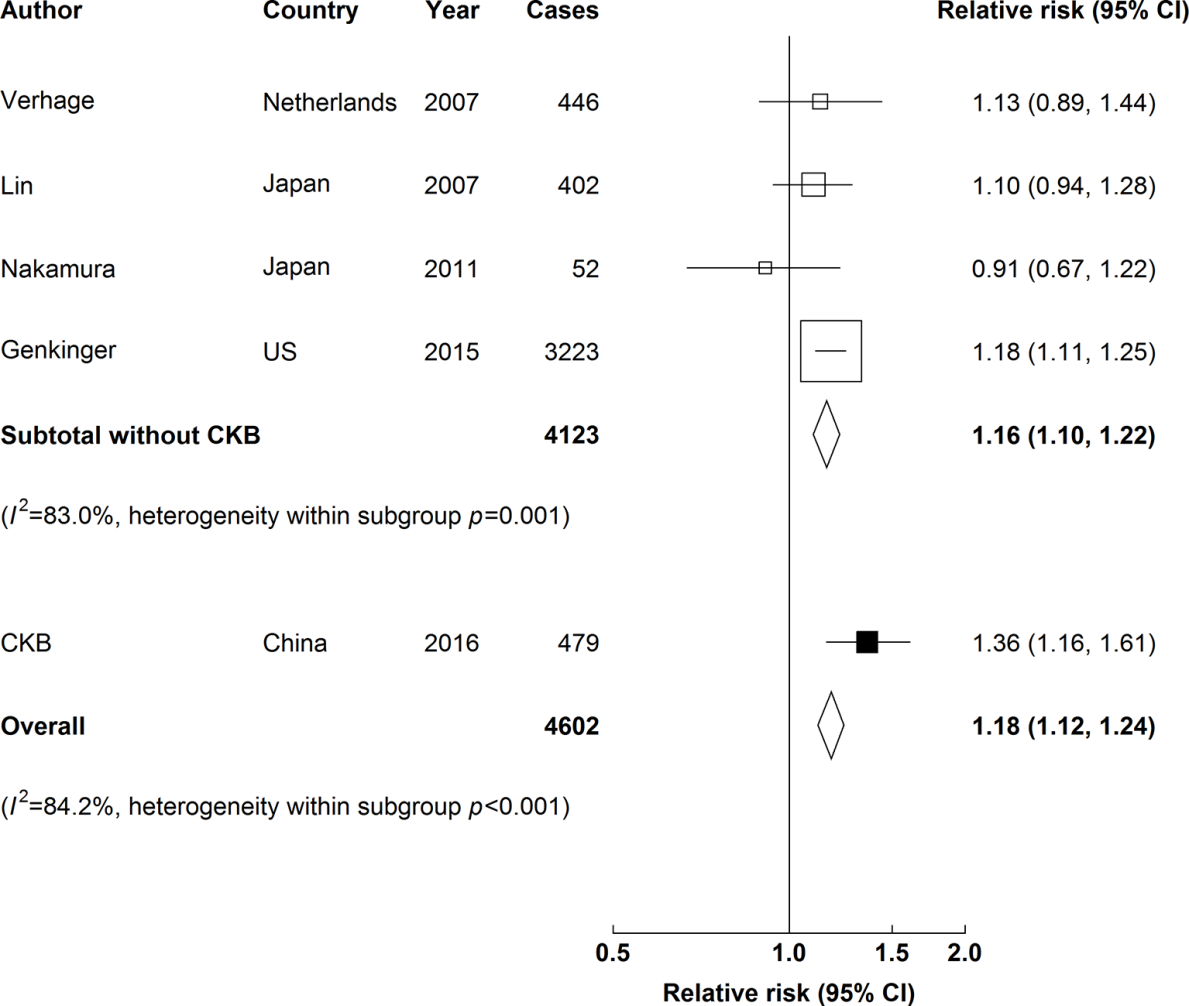
Adjusted RRs for pancreatic cancer associated with a 5 kg/m^2^ higher young adulthood BMI (weighted mean age of 20.9 years) in meta-analysis of CKB and four published studies. Boxes represent the RRs associated with a 5 kg/m^2^ higher BMI at young adulthood for individual studies, with the size of the box inversely proportional to the variance of the logRR. Open boxes represent previously published studies, and the black box represents CKB. Diamonds represent summary RRs. Within categories, RRs are ordered according to their year of publication. Estimates and 95% CI of the summary RRs are in bold. Heterogeneity between studies was assessed by *I*^2^ and Cochran’s *Q* test. BMI, body mass index; CKB, China Kadoorie Biobank; RRs, relative risks.

In meta-analysis of CKB and four other studies, young adulthood BMI was positively associated with risk of pancreatic cancer. For each 5 kg/m^2^ higher BMI, the overall adjusted HR was 1.18 (1.12 to 1.24) (figure 3), and the strength of the association was similar in Asia and in Western studies (online supplementary table 1). There was no evidence of publication bias (online supplementary figure 4).

**Figure 4:**
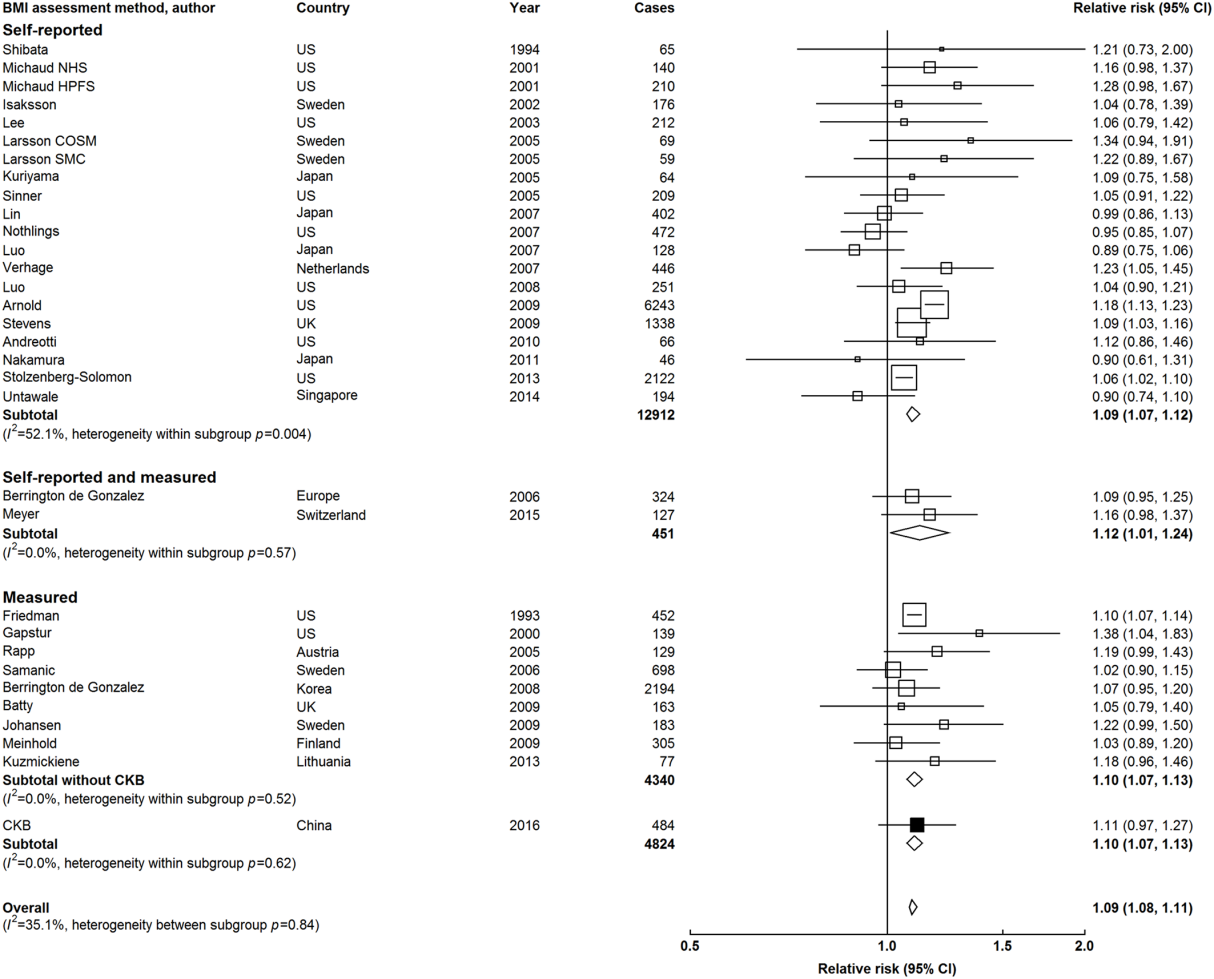
Adjusted RRs for pancreatic cancer associated with a 5 kg/m^2^ higher adulthood BMI (weighted mean age of 51.6 years) in meta-analysis of CKB and 31 published studies, stratified by BMI assessment methods. Conventions as in figure 3. BMI, body mass index; CKB, China Kadoorie Biobank; RRs, relative risks.

In meta-analysis of CKB with 31 other studies, adulthood BMI was positively associated with risk of pancreatic cancer. The strength of the association was somewhat weaker compared with that for young adulthood BMI, with adjusted overall RR of 1.09 (1.08 to 1.11) for a 5 kg/m^2^ higher level (figure 4). The overall risk estimates appeared to be similar, irrespective of how BMI was assessed. However, there was a large between-study heterogeneity in RRs among studies using self-reported BMI (*I*^2^=52.1%, Cochran’s *Q p*=0.004; figure 4), especially between Western and East Asian studies (online supplementary figure 5). In North America and Europe, there was a similarly strong positive association of pancreatic cancer with both self-reported and measured BMI. In contrast, there was no clear association of self-reported BMI with pancreatic cancer in East Asians, with RR of 0.94 (0.87 to 1.03), compared with RR of 1.08 (0.99 to 1.19) for CKB and one other East Asian study that used measured BMI (online supplementary figure 5). There was weak evidence of publication bias in studies conducted in East Asia (Egger’s test *p*=0.03) but not in North America or Europe (Egger’s test *p*=0.34, online supplementary figure 6). The results were similar when RR per one SD was used (online supplementary figure 7). Overall, the risk estimates did not appear to vary significantly by levels of population mean BMI or by median follow-up across different studies (*p* for heterogeneity=0.17 and 0.07, respectively, online supplementary figures 8 and 9). Likewise, there was little heterogeneity in risk estimates by sex, BMI assessment, exclusion of early periods, or different adjustment in the statistical models (online supplementary table 1). However, studies with higher mean age tended to show lower RRs (*p* for heterogeneity=0.47, online supplementary Figure 10).

Of the 31 studies included in the meta-analysis of adulthood BMI, three studies also reported associations with WC and WHR (online supplementary data). When they were combined with CKB, WC and WHR were each positively associated with risk of pancreatic cancer, with RRs for a 10 cm higher WC and a 0.1 higher WHR being 1.10 (1.06 to 1.14) and 1.14 (1.08 to 1.20), respectively. The associations attenuated slightly with further adjustment of adulthood BMI (CKB: HR 1.07 vs 1.02 per 10 cm higher WC) (online supplementary figure 11).

## Discussion

This is the first large prospective study of the association between adiposity and risk of pancreatic cancer in China. Among relatively lean Chinese adults, adulthood BMI and WC measured at around 50 years of age showed a positive association with risk of pancreatic cancer, while BMI at age 25 years was more strongly positively associated with pancreatic cancer later in life. The present study findings are largely consistent with previous published studies in Western populations as well as with more limited data in Eastern Asian populations, especially those using measured, rather than self-reported, BMI.

Previous prospective studies in Western populations have shown that both general and central adiposity in adulthood are associated with increased risks of pancreatic cancer.^8 10^ In contrast, 11 prospective cohort studies^17 18 22–30^ and two pooled analyses of prospective studies^31 32^ in East Asia have reported no such positive associations. In particular, the Shanghai Women’s Health Study identified 195 incident pancreatic cancer cases over a median of 15.1 years of follow-up, and they reported that neither BMI nor WHR was associated with risk of pancreatic cancer (HR per five units higher BMI 0.92 [0.74 to 1.14] and HR per SD higher WHR 1.08 [0.94 to 1.24]).^30^ Unlike CKB, most (five out of eight) East Asian studies used self-reported rather than measured BMI, which could result in measurement error and regression dilution bias; this is supported by the present meta-analyses that showed the association was stronger in East Asian studies using measured rather than self-reported BMI (HR 1.06 vs 0.94, respectively). However, we observed no similar difference in North American or European studies. East Asian studies using self-reported BMI did not differ from those using measured BMI in mean age, adjustments or mean follow-up periods (online supplementary table 3), although they tended to be smaller in size and not to exclude early years of follow-up,^26^ which may explain in part the observed heterogeneity. It is possible that individuals with undetected cancer at baseline may lose weight (ie, so-called reverse causality), resulting in a distorted association of BMI with pancreatic cancer. Additionally, the null association may reflect a chance finding or be due in part to publication bias. Though not significant, the risk estimates in CKB using properly measured adulthood BMI were in agreement with previous studies in North America and Europe (RR for a 5 kg/m^2^ increase 1.11 vs 1.11). Moreover, the mean BMI (~24 kg/m^2^) in East Asian studies was much lower than in Western studies (~28 kg/m^2^), and there is suggestive evidence that the strength of the association appeared to be somewhat weaker at low, than high, BMI in Western studies (online supplementary figure 8). We also showed a positive association between WC and risk of pancreatic cancer in a Chinese population, again consistent with evidence from Western populations (HR 1.07 vs 1.10), even though the association for WC in CKB attenuated with further adjustment for adulthood BMI, while the opposite was not seen.

Our meta-analysis on adulthood BMI was consistent with a previous meta-analysis by Aune *et al*.^10^ That meta-analysis included 23 prospective studies (9504 cases) for pancreatic cancer incidence and 7 prospective studies (8869 cases) for pancreatic cancer mortality and reported summary RRs per five units higher BMI of 1.10 (1.07 to 1.14, *I*^2^=19%) for pancreatic cancer incidence and of 1.16 (0.98 to 1.36, *I*^2^=56%) for pancreatic cancer mortality, with a similar association across Asian, North American and European studies. However, only three Asian studies were included, and the result was dominated by the study by Jee *et al*^17^ (2651 pancreatic cancer cases vs 288 in the other two studies). In addition, there was evidence of greater heterogeneity in studies using self-reported than in those using directly measured BMI. In our meta-analysis, we identified six studies set in Asian populations and showed that the association differed by the way in which BMI was assessed. Of note, for better comparability of the mean age with the other studies included in the meta-analysis (median 55.5), we substituted the study by Jee *et al* for that by Berrington de González *et al*,^18^ which used the same data. This has yielded summary RRs of 1.15 (1.08 to 1.22, *I*^2^=0%) for studies using measured BMI and of 1.08 (1.03 to 1.14, *I*^2^=68%) for all Asian studies.

Compared with adulthood BMI, fewer studies have previously assessed the associations of young adulthood BMI with risk of pancreatic cancer. In East Asia, only two previous studies with limited numbers of cases have assessed the association, with inconsistent findings.^25 28^ To our knowledge, CKB is the first large study in Asia reporting a positive association of young adulthood BMI with risk of pancreatic cancer. When meta-analysing CKB with previous studies, we confirmed the positive association between young adulthood BMI and risk of pancreatic cancer and that the associations did not differ significantly by regions. Compared with adult adiposity, the association for young adulthood adiposity and pancreatic cancer is less likely to be affected by bias from reverse causality. Pancreatic cancer has a long latency period, during which unintentional weight loss might occur as a result of subclinical disease.^33 34^ It has been suggested that it may take up to 20 years for pancreatic cancer to become clinically evident after the initial mutations.^35^ Therefore, excluding the first few years of follow-up might still be insufficient to fully account for reverse causality when assessing adulthood adiposity. Young adulthood BMI may mark developmental factors that lead to acquisition of both lean and fat mass in childhood and adolescence, which may influence later risk of cancer.^8^ In this context, adult height indicates developmental factors that lead to greater linear growth,^36 37^ while leg length is thought to mark the quality of the environment in early life.^38^ They might be also linked to cancer risk through similar mechanisms as young adulthood BMI, but these hypotheses were not fully supported by the CKB findings.

The strengths of the CKB include a prospective design, a large and diverse study population, the ability to assess a range of measures of both general and central adiposity and careful adjustment for other risk factors for pancreatic cancer. Moreover, the results of CKB were combined with published studies to facilitate direct comparison and pooled analyses. This study also has limitations. First, as in all previous studies, young adulthood BMI in CKB was calculated from self-reported weight assuming the same height as in adulthood. Such an approach might lead to overestimation of BMI as height generally decreases as people age.^39^ However, it is unlikely that the decrease in height with age differed by young adulthood BMI (or risk of pancreatic cancer). Moreover, there was good agreement in the weight recalled at different time points during the study, with the Pearson’s correlation coefficient being 0.81 for weight at age 25 years recalled at baseline survey and resurvey among the 19 788 participants who attended a resurvey 2 years after the baseline survey. Previous studies, including the Million Women Study and the Nurses’ Health Study, have also shown good agreement between directly measured and self-reported BMI.^10 11^ Second, as an observational study, our results do not necessarily indicate causality and residual confounding may still exist, especially that related to smoking.^40^ This is plausible as young adulthood BMI in the present study was positively associated with smoking at age 25 years, whereas the association was reversed for adulthood BMI (table 1). Thus, smoking could be a confounder of the association of young adulthood BMI (whereas it could represent a negative confounder of adulthood BMI). However, the associations were consistent in never smokers (online supplementary tables 5 and 6).

In summary, among Chinese adults, BMI in young adulthood was positively associated with risk of developing pancreatic cancer late in life, while adulthood BMI and WC also showed a suggestive positive association with pancreatic cancer. Our meta-analysis suggested that the association between measured adulthood BMI and pancreatic cancer in East Asian studies was consistent with North American or European studies. More large-scale prospective studies in Asia are needed to quantify reliably the association of adiposity and risk of pancreatic cancer. Elucidating the mechanisms that explain the association of adiposity with pancreatic cancer risk might contribute to understanding of the aetiology of this lethal cancer.

**What is already known on this subject**

Studies conducted in Western populations have concluded that higher adulthood adiposity is associated with an increased risk of pancreatic cancer. However, the association in Asian populations has been null. Moreover, evidence is limited for central adiposity in adulthood and adiposity in young adulthood.

**What this study adds**

In this Chinese population, we found that young adulthood body mass index (BMI) was strongly associated with risk of pancreatic cancer. Adulthood BMI and waist circumference also showed a positive association with pancreatic cancer, with the association less strong than young adulthood BMI. In meta-analyses, the association for adulthood BMI was similar between Western and East Asian studies that directly measured BMI, whereas the association was null in East Asian studies assessing BMI by self-report. Avoiding excess adiposity both in young adulthood and adulthood might prevent this fatal cancer.
