# Supplementary material for: Young adulthood and adulthood adiposity in relation to incidence of pancreatic cancer: a prospective study of 0.5 million Chinese adults and a meta-analysis
Source: J Epidemiol Community Health. 2017 Sep 12;71(11):1059–67. doi: 10.1136/jech-2017-208895 (PMC5847093; doi:10.1136/jech-2017-208895)

**Supplementary Material**

**Young adulthood and adulthood adiposity in relation to incidence of pancreatic cancer:**

**a prospective study of 0.5 million Chinese adults and a meta-analysis**

**Journal of Epidemiology and Community Health**

Yuanjie Pang^1^, ScM; Michael V Holmes^1,2,3^, MD, PhD; Christiana Kartsonaki^1,2^, DPhil; Yu Guo^4^, MSc; Ling Yang^1,2^, PhD; Zheng Bian^4^, MSc; Yiping Chen^1,2^, DPhil; Fiona Bragg^1^, DPhil; Andri Iona^1,2^, MSc; Iona Y Millwood^1,2^, DPhil; Junshi Chen^6^, MD;

Liming Li^4,5^, MD, MPH; Zhengming Chen^1^, DPhil

1. Clinical Trial Service Unit & Epidemiological Studies Unit (CTSU), Nuffield Department of Population Health, University of Oxford, Oxford, UK
2. Medical Research Council Population Health Research Unit (MRC PHRU) at the University of Oxford, Oxford, UK
3. National Institute for Health Research, Oxford Biomedical Research Centre, Oxford University Hospital, Oxford , UK
4. Chinese Academy of Medical Sciences, 9 Dongdan San Tiao, Beijing 100730, China
5. School of Public Health, Peking University, Beijing 100191, China
6. National Center for Food Safety Risk Assessment, 37 Guangqu Road, Beijing 100021, China

**Address for correspondence**

Michael V Holmes

MRC Population Health Research Unit at the University of Oxford

Big Data Institute Building

Old Road Campus

University of Oxford

Oxford, OX3 7LF, UK

Fax: 44-1865-743985

Email: michael.holmes@ndph.ox.ac.uk

Table of Contents

[Supplementary Data. Search strategies 3](#_Toc487128681)

[Supplementary Data. Studies included in meta-analysis 4](#_Toc487128682)

[Supplementary Data. Dose-response meta-analysis 7](#_Toc487128683)

[Supplementary Table 1. Adjusted RRs of PC for a 5 units higher young adulthood and adulthood BMI by various subgroups in meta-analysis 8](#_Toc487128684)

[Supplementary Table 2. Selected key characteristics of published prospective studies of young adulthood BMI and PC 9](#_Toc487128685)

[Supplementary Table 3. Selected key characteristics of published prospective studies of adulthood BMI and PC 11](#_Toc487128686)

[Supplementary Table 4. Adjusted HRs of PC by young adulthood BMI with additional adjustment for adulthood adiposity in CKB 19](#_Toc487128687)

[Supplementary Table 5. Adjusted HRs of PC by adulthood BMI stratified by smoking status in CKB 20](#_Toc487128688)

[Supplementary Table 6. Adjusted HRs of PC by young adulthood BMI stratified by smoking status in CKB 21](#_Toc487128689)

[Supplementary Table 7. Adjusted HRs for PC by BMI and WC in CKB 22](#_Toc487128690)

[Supplementary Table 8. Adjusted HRs for PC by tertiles of anthropometric measures at adulthood in CKB 23](#_Toc487128691)

[Supplementary Figure 1. PRISMA flow chart of literature search 24](#_Toc487128692)

[Supplementary Figure 2. Mean young adulthood and adulthood BMI by age at baseline in males and females in CKB 25](#_Toc487128693)

[Supplementary Figure 3. Adjusted HRs for PC by tertiles of anthropometric measures at adulthood in CKB 26](#_Toc487128694)

[Supplementary Figure 4. Funnel plot of published studies of young adulthood BMI and PC 27](#_Toc487128695)

[Supplementary Figure 5. Adjusted RRs for PC associated with a 5 units higher adulthood BMI, measured or self-reported, in meta-analysis of CKB and 31 published studies 28](#_Toc487128696)

[Supplementary Figure 6. Funnel plot of published studies of adulthood BMI and PC 29](#_Toc487128697)

[Supplementary Figure 7. Adjusted RRs for PC associated with 1 SD higher adulthood BMI, measured or self-reported, in meta-analysis of CKB and 31 published studies 30](#_Toc487128698)

[Supplementary Figure 8. Adjusted RRs for PC associated with a 5 units higher adulthood BMI in meta-analysis of CKB and 27 published studies, by study-level mean BMI 31](#_Toc487128699)

[Supplementary Figure 9. Adjusted RRs for PC associated with a 5 units higher adulthood BMI in meta-analysis of CKB and 31 published studies, by median follow-up (years) 32](#_Toc487128700)

[Supplementary Figure 10. Adjusted RRs for PC associated with a 5 units higher adulthood BMI in meta-analysis of CKB and 31 published studies, by mean age of each study 33](#_Toc487128701)

[Supplementary Figure 11. Adjusted RRs for PC associated with a 10 cm higher WC and a 0.1 higher WHR in meta-analysis of CKB and 3 published studies, with or without adjustment for BMI 34](#_Toc487128702)

# Supplementary Data. Search strategies

***Search 1: PubMed, inception through September 15, 2016***

(("Pancreatic Neoplasms"[Mesh]) OR

(pancreatic neoplas*[tiab] OR pancreas neoplas*) OR

(pancreatic cancer*[tiab] OR pancreas cancer*) OR

(pancreatic carcin*[tiab] OR pancreas carcin*) OR

(pancreatic tumo*[tiab] OR pancreas tumo*) OR

(pancreatic metasta*[tiab] OR pancreas metasta*) OR

(pancreatic malign*[tiab] OR pancreas malign*) OR

(pancreatic adenocarcinoma*[tiab] OR pancreas adenocarcinoma* )

AND ("Body Mass Index"[Mesh] OR body mass index*[tiab] OR BMI*[tiab]

"Obesity"[Mesh] OR obesity[tiab] OR obese[tiab]

"Overweight"[Mesh] OR overweight[tiab] OR

"Body Composition"[Mesh] OR body composition[tiab] OR DEXA[tiab] OR bioimpedence[tiab] OR

"Waist Circumference"[Mesh] OR waist circumference*[tiab] OR

"Hip Circumference"[Mesh] OR hip circumference*[tiab] OR

"Waist-Hip Ratio"[Mesh] OR waist hip ratio*[tiab] )

***Search 2: Embase, inception through September 15, 2016***

( pancreatic neoplasms/ OR

(pancreatic neoplas* OR pancreatic cancer* OR pancreatic carcin* OR pancreatic tumo* OR pancreatic metasta* OR pancreatic malign* OR pancreatic adenocarcinoma*).ti,ab. )

AND ( body mass index/ OR (body mass index* OR BMI*).ti,ab.

obesity/ OR (obesity OR obese).ti,ab. OR

overweight/ OR (overweight).ti,ab. OR

body composition/ OR (body composition OR DEXA OR bioimpedence).ti,ab. OR

waist circumference/ OR (waist circumference*).ti,ab. OR

hip circumference/ OR (hip circumference*).ti,ab. OR

waist-hip ratio/ OR (waist hip ratio*).ti,ab. )

# Supplementary Data. Studies included in meta-analysis

***Adulthood BMI (n=32):***

Friedman GD, Van den Eeden SK. Risk factors for pancreatic cancer: an exploratory study. *Int J Epidemiol* 1993;22(1):30-37.

Shibata A, Mack TM, Paganini‐Hill A, Ross RK, Henderson BE. A prospective study of pancreatic cancer in the elderly. *Int J Cancer* 1994;58(1):46-49.

Gapstur SM, Gann PH, Lowe W, Liu K, Colangelo L, Dyer A. Abnormal glucose metabolism and pancreatic cancer mortality. *JAMA* 2000;283(19):2552-8.

Michaud DS, Giovannucci E, Willett WC, Colditz GA, Stampfer MJ, Fuchs CS. Physical activity, obesity, height, and the risk of pancreatic cancer. *JAMA* 2001;286(8):921-9.

Isaksson B, Jonsson F, Pedersen NL, Larsson J, Feychting M, Permert J. Lifestyle factors and pancreatic cancer risk: a cohort study from the Swedish Twin Registry. *Int J Cancer* 2002;98(3):480-482.

Lee IM, Sesso HD, Oguma Y, Paffenbarger RS, Jr. Physical activity, body weight, and pancreatic cancer mortality. *Br J Cancer* 2003;88(5):679-83.

Kuriyama S, Tsubono Y, Hozawa A, Shimazu T, Suzuki Y, Koizumi Y, et al. Obesity and risk of cancer in Japan. *Int J Cancer* 2005;113(1):148-57.

Larsson SC, Permert J, Hakansson N, Naslund I, Bergkvist L, Wolk A. Overall obesity, abdominal adiposity, diabetes and cigarette smoking in relation to the risk of pancreatic cancer in two Swedish population-based cohorts. *Br J Cancer* 2005;93(11):1310-5.

Oh SW, Yoon YS, Shin SA. Effects of excess weight on cancer incidences depending on cancer sites and histologic findings among men: Korea National Health Insurance Corporation Study. *J Clin Oncol* 2005;23(21):4742-54.

Rapp K, Schroeder J, Klenk J, Stoehr S, Ulmer H, Concin H, et al. Obesity and incidence of cancer: a large cohort study of over 145,000 adults in Austria. *Br J Cancer* 2005;93(9):1062-7.

Sinner PJ, Schmitz KH, Anderson KE, Folsom AR. Lack of association of physical activity and obesity with incident pancreatic cancer in elderly women. *Cancer Epidemiol Biomarkers Prev* 2005;14(6):1571-1573.

Berringtong de González A, Spencer EA, Bueno-de-Mesquita HB, Roddam A, Stolzenberg-Solomon R, Halkjaer J, et al. Anthropometry, physical activity, and the risk of pancreatic cancer in the European prospective investigation into cancer and nutrition. *Cancer Epidemiol Biomarkers Prev* 2006;15(5):879-85.

Samanic C, Chow WH, Gridley G, Jarvholm B, Fraumeni Jr JF. Relation of body mass index to cancer risk in 362,552 Swedish men. *Cancer Causes Control* 2006;17(7):901-9.

Yun JE, Jo I, Park J, et al. Cigarette smoking, elevated fasting serum glucose, and risk of pancreatic cancer in Korean men. *Int J Cancer* 2006;119(1):208-12.

Lin Y, Kikuchi S, Tamakoshi A, Yagyu K, Obata Y, Inaba Y, et al. Obesity, physical activity and the risk of pancreatic cancer in a large Japanese cohort. *Int J Cancer* 2007;120(12):2665-71.

Luo J, Iwasaki M, Inoue M, Sasazuki S, Otani T, Ye W, et al. Body mass index, physical activity and the risk of pancreatic cancer in relation to smoking status and history of diabetes: a large-scale population-based cohort study in Japan-the JPHC study. *Cancer Causes Control* 2007;18(6):603-12.

Nothlings U, Wilkens LR, Murphy SP, Hankin JH, Henderson BE, Kolonel LN. Body mass index and physical activity as risk factors for pancreatic cancer: the Multiethnic Cohort Study. *Cancer Causes Control* 2007;18(2):165-75.

Verhage BA, Schouten LJ, Goldbohm RA, van den Brandt PA. Anthropometry and pancreatic cancer risk: an illustration of the importance of microscopic verification. *Cancer Epidemiol Biomarkers Prev* 2007;16(7):1449-54.

Berrington de González A, Yun JE, Lee SY, Klein AP, Jee SH. Pancreatic cancer and factors associated with the insulin resistance syndrome in the Korean cancer prevention study. *Cancer Epidemiol Biomarkers Prev* 2008;17(2):359-64.

Jee SH, Yun JE, Park EJ, Cho ER, Park IS, Sull JW, et al. Body mass index and cancer risk in Korean men and women. *Int J Cancer* 2008;123(8):1892-6.

Luo J, Margolis KL, Adami HO, LaCroix A, Ye W. Obesity and risk of pancreatic cancer among postmenopausal women: the Women's Health Initiative (United States). *Br J Cancer* 2008;99(3):527-31.

Inoue M, Noda M, Kurahashi N, Iwasaki M, Sasazuki S, Iso H, et al. Impact of metabolic factors on subsequent cancer risk: results from a large-scale population-based cohort study in Japan. *Eur J Cancer Prev* 2009;18(3):240-7.

Arnold LD, Patel AV, Yan Y, Jacobs EJ, Thun MJ, Calle EE, et al. Are racial disparities in pancreatic cancer explained by smoking and overweight/obesity? *Cancer Epidemiol Biomarkers Prev* 2009;18(9):2397-2405.

Batty GD, Kivimaki M, Morrison D, Huxley R, Smith GD, Clarke R, et al. Risk factors for pancreatic cancer mortality: extended follow-up of the original Whitehall Study. *Cancer Epidemiol Biomarkers Prev* 2009;18(2):673-5.

Johansen D, Borgstrom A, Lindkvist B, Manjer J. Different markers of alcohol consumption, smoking and body mass index in relation to risk of pancreatic cancer. A prospective cohort study within the Malmo Preventive Project. *Pancreatology* 2009;9(5):677-86.

Meinhold CL, de González AB, Albanes D, Weinstein SJ, Taylor PR, Virtamo J, et al. Predictors of fasting serum insulin and glucose and the risk of pancreatic cancer in smokers. *Cancer Causes Control* 2009;20(5):681-690.

Stevens RJ, Roddam AW, Spencer EA, Pirie KL, Reeves GK, Green J, et al. Factors associated with incident and fatal pancreatic cancer in a cohort of middle-aged women. *Int J Cancer* 2009;124(10):2400-5.

Andreotti G, Hou L, Freeman LE, Mahajan R, Koutros S, et al. Body mass index, agricultural pesticide use, and cancer incidence in the Agricultural Health Study cohort. *Cancer Causes Control* 2010;21(11):1759-75.

Nakamura K, Nagata C, Wada K, Tamai Y, Tsuji M, Takasuka N, et al. Cigarette smoking and other lifestyle factors in relation to the risk of pancreatic cancer death: a prospective cohort study in Japan. *Jpn J Clin Oncol* 2011;41(2):225-31.

Kuzmickiene I, Everatt R, Virviciute D, Tamosiunas A, Radisauskas R, Reklaitiene R, et al. Smoking and other risk factors for pancreatic cancer: a cohort study in men in Lithuania. *Cancer Epidemiol* 2013;37(2):133-9.

Stolzenberg-Solomon RZ, Schairer C, Moore S, Hollenbeck A, Silverman DT. Lifetime adiposity and risk of pancreatic cancer in the NIH-AARP Diet and Health Study cohort. Am *J Clin Nutri* 2013;98(4):1057-65.

Untawale S, Odegaard AO, Koh WP, Jin AZ, Yuan JM, Anderson KE. Body mass index and risk of pancreatic cancer in a Chinese population. *PLoS One* 2014;9(1):e85149.

Meyer J, Rohrmann S, Bopp M, Faeh D. Impact of Smoking and Excess Body Weight on Overall and Site-Specific Cancer Mortality Risk. *Cancer Epidemiol Biomarkers Prev* 2015;24(10):1516-22.

***Young adulthood BMI (n=4):***

Lin Y, Kikuchi S, Tamakoshi A, Yagyu K, Obata Y, Inaba Y, et al. Obesity, physical activity and the risk of pancreatic cancer in a large Japanese cohort. *Int J Cancer* 2007;120(12):2665-71.

Verhage BA, Schouten LJ, Goldbohm RA, van den Brandt PA. Anthropometry and pancreatic cancer risk: an illustration of the importance of microscopic verification. *Cancer Epidemiol Biomarkers Prev* 2007;16(7):1449-54.

Nakamura K, Nagata C, Wada K, Tamai Y, Tsuji M, Takasuka N, et al. Cigarette smoking and other lifestyle factors in relation to the risk of pancreatic cancer death: a prospective cohort study in Japan. *Jpn J Clin Oncol* 2011;41(2):225-31.

Genkinger JM, Kitahara CM, Bernstein L, Berrington de González A, Brotzman M, Elena JW, et al. Central adiposity, obesity during early adulthood, and pancreatic cancer mortality in a pooled analysis of cohort studies. *Ann Oncol* 2015;26(11):2257-66.

***Adulthood WC and WHR (n=3):***

Berringtong de González A, Spencer EA, Bueno-de-Mesquita HB, Roddam A, Stolzenberg-Solomon R, Halkjaer J, et al. Anthropometry, physical activity, and the risk of pancreatic cancer in the European prospective investigation into cancer and nutrition. *Cancer Epidemiol Biomarkers Prev* 2006;15(5):879-85.

Luo J, Margolis KL, Adami HO, LaCroix A, Ye W. Obesity and risk of pancreatic cancer among postmenopausal women: the Women's Health Initiative (United States). *Br J Cancer* 2008;99(3):527-31.

Genkinger JM, Spiegelman D, Anderson KE, Bernstein L, van den Brandt PA, Calle EE, et al. A pooled analysis of 14 cohort studies of anthropometric factors and pancreatic cancer risk. *Int J Cancer* 2011;129(7):1708-17.

# Supplementary Data. Dose-response meta-analysis

If a study did not report the relative risk (RR) per 5 kg/m^2^ higher BMI, we estimated it by quantifying the study-specific linear trends between exposure and outcome using the method described by Greenland and Longnecker [1].

For each of the included studies, we used the reported median or mean BMI to estimate RRs. For any study reporting only the range of BMI for a category, we used the mean value of the lower and upper bounds of that category. When the highest category was open-ended, its category BMI was calculated as the lower bound plus 1.5 times the width of the neighbouring category. Similar methods were used when the lowest category was open-ended. In studies that did not report the distribution of cases or person-years, we estimated these from the reported total number of cases and person-years [2].

**References**

1. Greenland S, Longnecker MP. Methods for trend estimation from summarized dose-response data, with applications to meta-analysis. *Am J Epidemiol* 1992; 135(11):1301-130.

2. Aune D, Greenwood DC, Chan DS, et al. Body mass index, abdominal fatness and pancreatic cancer risk: a systematic review and non-linear dose-response meta-analysis of prospective studies. *Ann Oncol* 2012;23(4):843-52.

# Supplementary Table 1. Adjusted RRs of PC for a 5 units higher young adulthood and adulthood BMI by various subgroups in meta-analysis

| **Subgroup** | **No. of studies** | **Fixed effects** | **Random effects** | ***I*^2^ (%)** | **Heterogeneity**  **within subgroup** | **Heterogeneity between subgroups** |
| --- | --- | --- | --- | --- | --- | --- |
|  |  | **RR (95% CI)** | **RR (95% CI)** |  |  |  |
| **Young adulthood BMI** |  |  |  |  |  |  |
| Overall | 5 | 1.18 (1.12, 1.24) | 1.16 (1.06, 1.27) | 84.2 | <0.01 | 0.92 |
| Region |  |  |  |  |  |  |
| Asia | 3 | 1.17 (1.05, 1.30) | 1.14 (0.92, 1.42) | 70.3 | 0.03 |  |
| North America or Europe | 2 | 1.18 (1.11, 1.25) | 1.18 (1.11, 1.25) | 0.0 | 0.74 |  |
| **Adulthood BMI** |  |  |  |  |  |  |
| Overall | 32 | 1.09 (1.08, 1.11) | 1.08 (1.05, 1.12) | 34.5 | 0.03 |  |
| Region |  |  |  |  |  | 0.06 |
| Asia | 7 | 1.01 (0.95, 1.08) | 1.01 (0.94, 1.08) | 9.5 | 0.36 |  |
| North America | 12 | 1.10 (1.08, 1.13) | 1.10 (1.05, 1.15) | 56.5 | 0.008 |  |
| Europe | 13 | 1.11 (1.06, 1.15) | 1.11 (1.06, 1.15) | 0.0 | 0.75 |  |
| North America or Europe | 25 | 1.10 (1.06, 1.12) | 1.10 (1.07, 1.14) | 28.9 | 0.09 |  |
| BMI assessment |  |  |  |  |  | 0.45 |
| Self-reported | 20 | 1.09 (1.07, 1.12) | 1.07 (1.02, 1.12) | 52.1 | 0.004 |  |
| Measured | 10 | 1.10 (1.07, 1.13) | 1.10 (1.07, 1.13) | 0.0 | 0.62 |  |
| Study-level mean BMI (kg/m^2^) |  |  |  |  |  | 0.17 |
| ≤24 | 8 | 1.01 (0.95, 1.08) | 1.01 (0.95, 1.08) | 0.0 | 0.46 |  |
| 24 – 26 | 12 | 1.10 (1.05, 1.16) | 1.13 (1.05, 1.21) | 35.6 | 0.11 |  |
| 26 – 28 | 8 | 1.11 (1.08, 1.13) | 1.10 (1.04, 1.15) | 56.1 | 0.03 |  |
| Median follow-up (years) |  |  |  |  |  |  |
| 6 – 8 | 9 | 1.07 (1.03, 1.12) | 1.07 (1.02, 1.12) | 2.8 | 0.41 | 0.07 |
| 8 – 12 | 6 | 1.05 (1.01, 1.09) | 1.04 (0.98, 1.11) | 23.0 | 0.26 |  |
| 12 – 18 | 8 | 1.09 (1.06, 1.13) | 1.09 (1.04, 1.14) | 16.9 | 0.30 |  |
| 18 + | 9 | 1.16 (1.12, 1.21) | 1.16 (1.12, 1.21) | 0.0 | 0.50 |  |
| Exclusion of early periods |  |  |  |  |  | 0.51 |
| No | 23 | 1.09 (1.06, 1.11) | 1.09 (1.06, 1.11) | 8.6 | 0.34 |  |
| Yes | 9 | 1.11 (1.08, 1.14) | 1.10 (1.04, 1.17) | 63.6 | 0.005 |  |
| Adjustment for smoking |  |  |  |  |  | 0.84 |
| No | 1 | 1.12 (0.86, 1.63) | 1.12 (0.86, 1.46) | – | – |  |
| Yes | 30 | 1.09 (1.08, 1.11) | 1.08 (1.05, 1.12) | 37.1 | 0.02 |  |
| Adjustment for alcohol |  |  |  |  |  | 0.65 |
| No | 23 | 1.10 (1.08, 1.12) | 1.09 (1.06, 1.12) | 34.5 | 0.05 |  |
| Yes | 8 | 1.07 (0.99, 1.15) | 1.07 (0.97, 1.18) | 39.7 | 0.11 |  |
| Adjustment for diabetes |  |  |  |  |  | 0.78 |
| No | 4 | 1.07 (1.00, 1.16) | 1.07 (1.00, 1.16) | 0.0 | 0.94 |  |
| Yes | 4 | 1.05 (0.98, 1.12) | 1.05 (0.98, 1.12) | 0.0 | 0.91 |  |

Heterogeneity between subgroups was calculated from meta-regression analysis.

# Supplementary Table 2. Selected key characteristics of published prospective studies of young adulthood BMI and PC

| **Author, publication year, country/ region** | **Study name** | **Follow-up period, median/mean** | **Study size, number of cases** | **Assessment of weight and height** | **BMI measure** | **Description of categories** | **RR (95% CI)** | **Adjustment for confounders** |
| --- | --- | --- | --- | --- | --- | --- | --- | --- |
| Genkinger， 2015, US | National Cancer Institute BMI and Mortality Cohort Consortium | 1980-2012, 3-26 years | 1359149, 3223 cases | Self-reported | BMI at 18-21 years | 15-18.4  18.5-20.9  21-22.9  23-24.9  25-27.4  27.5-29.9  30-39.9  Per 5 units | 1.01 (0.89, 1.14)  0.98 (0.89, 1.08)  1.00  1.13 (1.01, 1.26)  1.36 (1.20, 1.55)  1.48 (1.20, 1.84)  1.43 (1.11, 1.85)  1.18 (1.11, 1.25) | Age, race, smoking, alcohol, cohort, education, marital, overall physical activity level, BMI at baseline |
| Nakamura, 2011, Japan | Takayama Study | 1992-1999, 6.7 years | 31552,  52 cases | Self-reported | BMI at 20 years in women  BMI at 20 years in men | <20.0  20.0-22.0  >22.0  <21.3  21.3-23.6  >23.6 | 1.00  0.70 (0.19, 2.64)  1.05 (0.33, 3.36)  1.00  0.41 (0.14, 1.18)  0.71 (0.31, 1.62) | Age, smoking, diabetes |
| Lin, 2007, Japan | Japanese Collaborative Cohort study for Evaluation of Cancer Risk | 1988-2003, 11.8 years | 110792,  402 cases | Self-reported | BMI at 20 years in women  BMI at 20 years in men | <20  20-22.4  22.5-24.9  25.0-27.4  27.5-29.9  ≥30  <20  20-22.4  22.5-24.9  25.0-27.4  27.5-29.9  ≥30 | 0.81 (0.50, 1.31)  1.00  1.08 (0.73, 1.61)  0.69 (0.39, 1.23)  0.46 (0.14, 1.48)  0.43 (0.06, 3.15)  1.39 (0.86, 2.24)  1.00  1.13 (0.75, 1.71)  1.54 (0.92, 2.58)  1.65 (0.70, 3.86)  3.51 (1.26, 9.78) | Age, smoking, diabetes, history of gallbladder diseases |

| **Author, publication year, country/ region** | **Study name** | **Follow-up period, median/mean** | **Study size, number of cases** | **Assessment of weight and height** | **BMI measure** | **Description of categories** | **RR (95% CI)** | **Adjustment for confounders** |
| --- | --- | --- | --- | --- | --- | --- | --- | --- |
| Verhage, 2007, Netherlands | Netherlands Cohort Study | 1986-1999, 13.3 years | 446 cases 4774 subcohort members, 446 cases | Self-reported | BMI at 20 years in women  BMI at 20 years in men | <20  20-20.9  21-22.9  ≥23  Per 1 unit  <20  20-20.9  21-22.9  ≥23  Per 1 unit | 1.00  0.93 (0.58, 1.51)  0.69 (0.46, 1.04)  0.97 (0.66, 1.44)  1.03 (0.96, 1.10)  1.00  0.80 (0.46, 1.40)  0.99 (0.62, 1.59)  1.07 (0.67, 1.73)  1.02 (0.95, 1.09) | Age, smoking, history of diabetes and hypertension |

# Supplementary Table 3. Selected key characteristics of published prospective studies of adulthood BMI and PC

| **Author, publication year, country/ region** | **Study name** | **Follow-up period, median/mean** | **Study size, age, number of cases** | **Exclusion of first few years of follow-up** | **Assessment of weight and height** | **Participants (all individuals / subgroups)** | **Description of categories** | **RR (95% CI)** | **Adjustment for confounders** |
| --- | --- | --- | --- | --- | --- | --- | --- | --- | --- |
| Meyer, 2015, Switzerland | Pooled analysis of 4 cohorts in Sweden | 1977-2008, 18.9 years | 35784, age 14-99 years: 127 cases | NA | Measured and self-reported | All individuals | <25  25-29.9  ≥30 | 1.00  1.20 (0.81, 1.78)  1.60 (0.93, 2.75) | Age, sex, smoking, alcohol, survey, physical activity, civil status, years of education, nationality, healthy diet |
| Untawale, 2014, Singapore | Singapore Chinese Health Study | 1993-2011, 14.8 years | 51251, age 45-74 years: 194 cases | 3 years | Self-reported | All individuals | <18.5  18.5-21.4  21.5-24.4  24.5-27.4  ≥27.5 | 1.83 (1.08, 3.09)  1.21 (0.81, 1.82)  1.00  1.31 (0.85, 2.00)  1.13 (0.64, 2.00) | Age, sex, smoking, alcohol, year of enrolment, dialect, education, diabetes, dietary pattern score, physical activity, sleep, energy intake |
| Kuzmickiene, 2013, Lithuania | Kaunas-Rotterdam Intervention Study and Multifactorial Ischemic Heart Disease Prevention Study | 1980-2008, 19.3 years | 7132, mean age 52.6 years: 77 cases | 3 years | Measured | All individuals | <25  25-29.9  ≥30 | 1.00  0.96 (0.55, 1.69)  1.66 (0.88, 3.15) | Age, smoking, alcohol, education |
| Stolzenberg-Solomon, 2013, US | NIH-AARP Diet and Health Study | 1995-2006, 10.5 years | 501698, age 50-71 years: 2122 cases | 1 year | Self-reported | All individuals | <18.5  18.5-24.9  25.0-29.9  30-34.9  ≥35 | 1.18 (0.79, 1.75)  1.00  1.09 (0.98, 1.20)  1.14 (1.00, 1.30)  1.29 (1.07, 1.55) | Age, sex, smoking, energy, energy-adjusted total fat |

| **Author, publication year, country/ region** | **Study name** | **Follow-up period, median/mean** | **Study size, age, number of cases** | **Exclusion of first few years of follow-up** | **Assessment of weight and height** | **Participants (all individuals / subgroups)** | **Description of categories** | **RR (95% CI)** | **Adjustment for confounders** |
| --- | --- | --- | --- | --- | --- | --- | --- | --- | --- |
| Nakamura, 2011, Japan | Takayama Study | 1992-1999, 6.7 years | 31552, mean age 54.7 (M) 55.8 (F), 52 cases | NA | Self-reported | Women  Men | ≤20.7  20.8-22.9  ≥23.0  ≤21.3  21.4-23.5  ≥23.6 | 1.00  0.37 (0.08, 1.77)  1.42 (0.52, 3.85)  1.00  0.55 (0.23, 1.32)  0.59 (0.23, 1.50) | Age, smoking, diabetes |
| Andreotti, 2010, US | Agricultural Health Study | 1993-2005, 10 years | 67947, age <40-70 years: 66 cases | NA | Self-reported | Women  Men | 18.5-24.9  25-29.9  30-34.9  Per 1 unit  18.5-24.9  25-29.9  30-34.9  Per 1 unit | 1.00  1.30 (0.43, 3.90)  2.48 (0.79, 7.83)  1.06 (0.98, 1.15)  1.00  1.11 (0.54, 2.27)  1.06 (0.42, 2.65)  0.99 (0.92, 1.07) | Age, race, diabetes |
| Arnold, 2009, US | American Cancer Prevention Study II | 1984-2004, 20 years | 1.2 millions, median age 57 years:6243 cases | 2 years | Self-reported | Blacks  Whites | <18.5  18.5-24.9  25-29.9  ≥30  <18.5  18.5-24.9  25-29.9  ≥30 | 0.44 (0.11, 1.77)  1.00  0.89 (0.70, 1.14)  1.06 (0.80, 1.42)  0.93 (0.75, 1.16)  1.00  1.15 (1.08, 1.22)  1.40 (1.28, 1.52) | Stratified by age at enrolment and adjusted for sex, smoking, diabetes, family history of pancreatic cancer, cholecystectomy |
| Batty, 2009, UK | Whitehall Study | 1967-2000, 38 years | 17898 men, age 40-69 years: 163 cases | NA | Measured | All individuals | Tertile 1  Tertile 2  Tertile 3  Per 2.98 units | 1.00  1.02 (0.69, 1.50)  1.18 (0.79, 1.75)  1.03 (0.87, 1.23) | Age, smoking, plasma cholesterol, physical activity, socioeconomic status, diabetes/blood glucose, marital status, forced expiratory volume in 1 s, height, DBP, SBP |

| **Author, publication year, country/ region** | **Study name** | **Follow-up period, median/mean** | **Study size, age, number of cases** | **Exclusion of first few years of follow-up** | **Assessment of weight and height** | **Participants (all individuals / subgroups)** | **Description of categories** | **RR (95% CI)** | **Adjustment for confounders** |
| --- | --- | --- | --- | --- | --- | --- | --- | --- | --- |
| Johansen, 2009, Sweden | Malmo Preventive Project | 1974-2004, 22.1 years | 33346, mean age 50 (M) 44 (F), 183 cases | NA | Measured | All individuals | <20  20-24.9  25-29.9  ≥30 | 0.84 (0.44, 1.61)  1.00  0.83 (0.60, 1.16)  1.38 (0.83, 2.28) | Age, sex, smoking |
| Meinhold, 2009, Finland | ATBC Cancer Prevention Study | 1985-2004, 16 years | 27035 men, age 50-69 years; 305 cases | 5 years | Measured | All individuals | <25  25-29.9  30-34.9  ≥35 | 1.00  0.97 (0.76, 1.24)  1.03 (0.72, 1.47)  1.42 (0.69, 2.93) | Age, smoking, BMI, total calories |
| Stevens, 2009, UK | Million Women Study | 1996-2007, 7.2 years | 1.3 millions women, mean age 55.9 years: 1338 cases | NA | Self-reported | All individuals | <22.5  22.5-24.9  25-27.4  27.5-29.9  30-32.4  ≥32.5 | 1.02 (0.88, 1.16)  1.00 (0.89, 1.12)  0.99 (0.88, 1.11)  1.17 (0.98, 1.40)  1.27 (1.00, 1.61)  1.42 (1.12, 1.80) | Age, smoking, region, socioeconomic status |
| Berrington de González, 2008, Korea | Korean Cancer Prevention Study | 1992-2006, 12 years | 631172, mean age: 55.8, 2194 cases | 2 years | Measured | All individuals | <18.5  18.5-22.9  23.0-27.4  >27.5 | 0.78 (0.60, 1.00)  1.00  0.98 (0.89, 1.07)  0.95 (0.80, 1.12) | Age, gender, smoking, fasting serum glucose |
| Luo, 2008, US | Women's Health Initiative | 1993-2005, 7.7 years | 161808 women, age 50-79 years: 251 cases | 2 years | Self-reported | All individuals | <22  22-24.9  25-29.9  30-34.9  ≥35 | 0.8 (0.5, 1.2)  1.0  0.9 (0.6, 1.2)  1.1 (0.8, 1.6)  0.9 (0.5, 1.4) | Age, smoking, different treatment assignments in clinical trials |

| **Author, publication year, country/ region** | **Study name** | **Follow-up period, median/mean** | **Study size, age, number of cases** | **Exclusion of first few years of follow-up** | **Assessment of weight and height** | **Participants (all individuals / subgroups)** | **Description of categories** | **RR (95% CI)** | **Adjustment for confounders** |
| --- | --- | --- | --- | --- | --- | --- | --- | --- | --- |
| Lin, 2007, Japan | Japanese Collaborative Cohort study for Evaluation of Cancer Risk | 1988-2003, 11.8 years | 110792, age 40-79 years: 402 cases | 3 years | Self-reported | Women  Men | <20  20-22.4  22.5-24.9  25.0-27.4  27.5-29.9  ≥30  <20  20-22.4  22.5-24.9  25.0-27.4  27.5-29.9  ≥30 | 1.15 (0.74, 1.80)  1.00  1.33 (0.91, 1.95)  1.21 (0.77, 1.92)  1.57 (0.86, 2.86)  1.04 (0.37, 2.89)  1.12 (0.76, 1.63)  1.00  0.94 (0.66, 1.34)  1.02 (0.65, 1.62)  0.62 (0.23, 1.70)  0.58 (0.08, 4.16) | Age, smoking, diabetes, a history of gallstones/cholecystitis |
| Luo, 2007, Japan | Japanese Public Health Center Study | 1990-2003, 11 years | 140420, age 30-79 years: 224 cases | 4 years | Self-reported | All individuals | <21  21-24.9  25-40 | 1.6 (1.0, 2.6)  1.0  0.7 (0.4, 1.2) | Age, smoking, alcohol, diabetes, BMI, leisure-time PA, study area, history of cholelithiasis |
| Nothlings, 2007, US | Multiethnic Cohort Study | 1993-2002, 7.5 years | 167430, age 45-75 years: 472 cases | 2 years | Self-reported | Women  Men | <25  25-29.9  ≥30  <25  25-29.9  ≥30 | 1.0  0.80 (0.59, 1.09)  0.65 (0.43, 0.99)  1.0  0.99 (0.74, 1.33)  1.51 (1.02, 2.26) | Age, ethnicity, smoking, family history of pancreatic cancer, history of diabetes mellitus, energy intake, red meat, processed meat, physical activity |

| **Author, publication year, country/ region** | **Study name** | **Follow-up period, median/mean** | **Study size, age, number of cases** | **Exclusion of first few years of follow-up** | **Assessment of weight and height** | **Participants (all individuals / subgroups)** | **Description of categories** | **RR (95% CI)** | **Adjustment for confounders** |
| --- | --- | --- | --- | --- | --- | --- | --- | --- | --- |
| Verhage, 2007, Netherlands | Netherlands Cohort Study | 1986-1999, 13.3 years | 4774 subcohort members, age 55-69 years: 446 cases | 2 years | Self-reported | Women  Men | <23  23-24.9  25-26.9  27-29.9  ≥30  Per 1 unit  <23  23-24.9  25-26.9  27-29.9  ≥30  Per 1 unit | 1.02 (0.66, 1.58)  1.00  1.69 (1.11, 2.58)  1.41 (0.89, 2.25)  1.31 (0.74, 2.31)  1.04 (1.00, 1.08)  1.10 (0.72, 1.69)  1.00  0.93 (0.61, 1.39)  1.17 (0.75, 1.81)  2.69 (1.47, 4.92)  1.05 (0.99, 1.12) | Age, smoking, history of diabetes and hypertension |
| Berrington de González, 2006, Europe | European Prospective Investigation into Cancer and Nutrition | 1991-2004, 6 years | 438405, age 19-84 years: 324 cases | 2 years | Self-reported and measured | All individuals | <20  20-22.9  23-24.9  25-26.9  27-29.9  30-34.9  ≥35  per 5 units | 0.55 (0.23, 1.31)  1.00  0.87 (0.58, 1.31)  0.74 (0.49, 1.14)  0.75 (0.46, 1.24)  1.10 (0.69, 1.76)  1.22 (0.60, 2.49)  1.14 (0.97, 1.33) | Age, sex, smoking, country, diabetes |
| Samanic, 2006, Sweden | Swedish Construction Worker's Study | 1971-1999, 19 years follow-up | 362552 men, age 18-67 years: 698 cases | NA | Measured | All individuals | <25  25-29.9  ≥30 | 1.00  0.95 (0.82, 1.12)  1.16 (0.87, 1.53) | Age, smoking, calendar year, DBP |
| Kuriyama, 2005, Japan | Miyagi Prefecture Cohort Study | 1984-1992, 9 years | 33453, age ≥40 years: 64 cases | NA | Self-reported | Women  Men | 18.5-24.9  25.0-27.4  ≥27.5  18.5-24.9  25.0-27.4  ≥27.5 | 1.00  0.63 (0.22, 1.83)  1.41 (0.56, 3.51)  1.00  0.40 (0.10, 1.72)  1.38 (0.40, 4.73) | Age, smoking, alcohol, meat, fish, fruits, green or yellow vegetables, bean-paste soup, type of health insurance |

| **Author, publication year, country/ region** | **Study name** | **Follow-up period, median/mean** | **Study size, age, number of cases** | **Exclusion of first few years of follow-up** | **Assessment of weight and height** | **Participants (all individuals / subgroups)** | **Description of categories** | **RR (95% CI)** | **Adjustment for confounders** |
| --- | --- | --- | --- | --- | --- | --- | --- | --- | --- |
| Larsson, 2005, Sweden | Swedish Mammograph-y Cohort and Cohort of Swedish Men | 1997-2004, 6.8 years | 83053, mean age 62 (F) 60 (M); 136 cases | 1 year | Self-reported | Women  Men | <20  20-24.9  25-29.9  ≥30  Per 1 unit  <20  20-24.9  25-29.9  ≥30  Per 1 unit | 0.76 (0.28, 2.59)  1.00  1.57 (0.87, 2.81)  1.48 (0.60, 3.62)  1.04 (0.97, 1.11)  1.54 (0.35, 6.66)  1.00  1.06 (0.62, 1.82)  2.08 (1.02, 4.25)  1.06 (0.99, 1.14) | Age, smoking, alcohol, education, physical activity, height |
| Oh, 2005, Korea | Korea National Health Insurance Corporation | 1992-2001, 10 years | 781283, age ≥20 years: 466 cases | NA | Measured | All individuals | <18.5  18.5-22.9  23.0-24.9  25.0-26.9  27.0-29.9  ≥30 | 0.53 (0.22, 1.29)  1.00  1.07 (0.86, 1.32)  0.87 (0.67, 1.15)  0.78 (0.51, 1.18)  1.04 (0.39, 2.81) | Age, smoking, alcohol , physical activity, family history of cancer, residency area |
| Rapp, 2005, Austria | Vorarlberg Health Monitoring and Promotion Program | 1985-2002, 9.9 years | 167371, mean age 42 years: 129 cases | 1 year | Measured | Women  Men | 18.5-24.9  25-29.9  ≥30  18.5-24.9  25-29.9  ≥30 | 1.00  0.87 (0.49, 1.53)  1.42 (0.76, 2.68)  1.00  1.29 (0.73, 2.37)  2.34 (1.17, 4.66) | Age, smoking, occupation group |
| Sinner, 2005, US | Iowa Women's Health Study | 1986-2001, 15 years | 38002 women, age 55-69 years;  209 cases | 2 years | Self-reported | All individuals | <25  25-29.9  ≥30 | 1.00  0.94 (0.69, 1.29)  1.14 (0.81, 1.62) | Age, smoking, multivitamin use |
| Lee, 2003, US | College Alumni Health Study | 1962-1995, 34 years | 32687, mean age: 48 years; 212 cases | NA | Self-reported | All individuals | <22.5  22.5-24.9  25-27.4  ≥27.5 | 1.00  0.84 (0.59, 1.22)  1.08 (0.59, 1.22)  0.99 (0.60, 1.62) | Age, sex, smoking, diabetes, physical activity |

| **Author, publication year, country/ region** | **Study name** | **Follow-up period, median/mean** | **Study size, age, number of cases** | **Exclusion of first few years of follow-up** | **Assessment of weight and height** | **Participants (all individuals / subgroups)** | **Description of categories** | **RR (95% CI)** | **Adjustment for confounders** |
| --- | --- | --- | --- | --- | --- | --- | --- | --- | --- |
| Isaksson, 2002, Sweden | Swedish Twin Registry Cohort | 1969-1997, 16 years | 21884, median age 56 years; 176 cases | NA | Self-reported | All individuals | <18.5  18.5-24.9  25-29.9  ≥30 | 2.30 (0.93, 5.71)  1.00  1.36 (0.99, 1.88)  0.56 (0.20, 1.52) | Age, sex, smoking |
| Michaud, 2001, US | Nurses’ Health Study 1 and Health Professionals Follow-up Study | 1976-1998, 20 years | 117041 women aged 30-55 years, 210 cases; 46648 men aged 40-75 years, 140 cases | 4 years | Self-reported | Women  Men | <23  23-24.9  25-26.9  27-29.9  ≥30  <23  23-24.9  25-26.9  27-29.9  ≥30 | 1.00  1.09 (0.79, 1.49)  1.29 (0.92, 1.80)  1.30 (0.91, 1.87)  1.72 (1.19, 2.48)  1.00  1.09 (0.79, 1.49)  1.29 (0.92, 1.80)  1.30 (0.91, 1.87)  1.72 (1.19, 2.48) | Age, smoking, height, diabetes |
| Gapstur, 2000, US | Chicago Heart Association Detection Project | 1963-1995, 25 years | 35658, age 15-90 years: 139 cases | NA | Measured | Women  Men | ≤22.98  22.99-23.24  23.24-26.16  ≥26.16  ≤24.13  24.14-26.29  26.30-28.63  ≥28.63 | 1.00  0.48 (0.17, 1.36)  1.09 (0.47, 2.51)  0.73 (0.30, 1.80)  1.00  1.76 (0.83, 3.74)  1.68 (0.80, 3.53)  3.04 (1.52, 6.08) | Age, smoking, postload plasma glucose |

| **Author, publication year, country/ region** | **Study name** | **Follow-up period, median/mean** | **Study size, age, number of cases** | **Exclusion of first few years of follow-up** | **Assessment of weight and height** | **Participants (all individuals / subgroups)** | **Description of categories** | **RR (95% CI)** | **Adjustment for confounders** |
| --- | --- | --- | --- | --- | --- | --- | --- | --- | --- |
| Shibata, 1994, US | Leisure World Cohort Study | 1981-1990, 7.2 years | 13976, mean age 75 years; 65 cases | NA | Self-reported | All individuals | Tertile 1  Tertile 2  Tertile 3 | 1.00  0.96 (0.50, 1.84)  1.23 (0.66, 2.28) | Age, sex, smoking |
| Friedman, 1993, US | Multiphase Check-up Study | 1964-1988, 12 years | 779 cases 2687 controls, age 15-94 years | NA | Measured | All individuals | Per 1 unit | 1.02 (1.00, 1.04) | Age, sex, smoking, examination site, date of first check-up, race |

F, female; M, male; NA, data not applicable.

# Supplementary Table 4. Adjusted HRs of PC by young adulthood BMI with additional adjustment for adulthood adiposity in CKB

|  | **HR (95% CI)** |
| --- | --- |
|  |  |
| **Basic adjustment** |  |
| <20.0 | 0.78 (0.61, 0.99) |
| 20.0-22.4 | 1.00 (0.86, 1.16) |
| 22.5-24.9 | 1.07 (0.91, 1.26) |
| 25.0-26.9 | 1.42 (1.10, 1.83) |
| ≥27.0 | 1.49 (1.05, 2.11) |
| per 5 kg/m^2^ | 1.36 (1.16, 1.61) |
|  |  |
| **+ WC** |  |
| <20.0 | 1.29 (1.11, 1.50) |
| 20.0-22.4 | 1.00 (0.78, 1.28) |
| 22.5-24.9 | 1.39 (1.18, 1.64) |
| 25.0-26.9 | 1.81 (1.41, 2.33) |
| ≥27.0 | 1.92 (1.36, 2.72) |
| per 5 kg/m^2^ | 1.36 (1.15, 1.60) |
|  |  |
| **+ HC** |  |
| <20.0 | 1.29 (1.11, 1.50) |
| 20.0-22.4 | 1.00 (0.78, 1.28) |
| 22.5-24.9 | 1.39 (1.18, 1.64) |
| 25.0-26.9 | 1.81 (1.41, 2.33) |
| ≥27.0 | 1.92 (1.36, 2.72) |
| per 5 kg/m^2^ | 1.36 (1.15, 1.60) |
|  |  |
| **+ WHR** |  |
| <20.0 | 1.29 (1.11, 1.50) |
| 20.0-22.4 | 1.00 (0.78, 1.27) |
| 22.5-24.9 | 1.40 (1.18, 1.64) |
| 25.0-26.9 | 1.81 (1.41, 2.34) |
| ≥27.0 | 1.93 (1.36, 2.73) |
| per 5 kg/m^2^ | 1.36 (1.15, 1.60) |
|  |  |
| **+ Body fat percentage** |  |
| <20.0 | 1.30 (1.12, 1.51) |
| 20.0-22.4 | 1.00 (0.78, 1.28) |
| 22.5-24.9 | 1.41 (1.19, 1.66) |
| 25.0-26.9 | 1.84 (1.43, 2.37) |
| ≥27.0 | 1.97 (1.39, 2.79) |
| per 5 kg/m^2^ | 1.37 (1.16, 1.62) |

For basic adjustment, the model was stratified by age at risk, sex, and region, and adjusted for education, smoking, and alcohol.

# Supplementary Table 5. Adjusted HRs of PC by adulthood BMI stratified by smoking status in CKB

| **BMI measure** | | **No. events** | | **No. people** | **Rate** | **HR (95% CI)** |
| --- | --- | --- | --- | --- | --- | --- |
|  | |  | |  | per 100,000 |  |
| **Never regular smokers** | | | |  |  |  |
| <20.0 | 7 | | | 12562 | 55.73 | 0.58 (0.28, 1.23) |
| 20.0-23.4 | 93 | | | 120832 | 76.99 | 1.00 (0.81, 1.24) |
| 23.5-26.9 | 107 | | | 135613 | 78.91 | 0.93 (0.77, 1.12) |
| ≥27.0 | 50 | | | 44514 | 112.32 | 1.14 (0.85, 1.52) |
| Total | 257 | | | 313521 | 81.97 | – |
| per 1 SD | – | | | – | – | 1.09 (0.97, 1.23) |
| per 5 kg/m^2^ | – | | | – | – | 1.14 (0.95, 1.36) |
| **Current regular smokers** | | | |  |  |  |
| <20.0 | | | 67 | 59969 | 111.7 | 0.97 (0.76, 1.24) |
| 20.0-23.4 | | | 31 | 24477 | 126.6. | 1.00 (0.69, 1.45) |
| 23.5-26.9 | | | 59 | 46086 | 128.0 | 1.11 (0.86, 1.44) |
| ≥27.0 | | | 21 | 19588 | 107.2 | 0.97 (0.63, 1.51) |
| Total | | | 178 | 150120 | 118.6 | – |
| per 1 SD | | | – | – | – | 1.01 (0.85, 1.18) |
| per 5 kg/m^2^ | | | – | – | – | 1.01 (0.79, 1.28) |

The models were stratified by age at risk, sex, and region, and adjusted for education, alcohol, and adulthood BMI. SD for adulthood BMI was 3.42 kg/m^2^. *P* for heterogeneity = 0.43.

# Supplementary Table 6. Adjusted HRs of PC by young adulthood BMI stratified by smoking status in CKB

| **BMI measure** | | **No. events** | | **No. people** | **Rate** | **HR (95% CI)** |
| --- | --- | --- | --- | --- | --- | --- |
|  | |  | |  | per 100,000 |  |
| **Not smoking at age 25** | | | |  |  |  |
| <20.0 | 169 | | | 160203 | 105.5 | 0.68 (0.51, 0.91) |
| 20.0-23.4 | 47 | | | 76411 | 61.5 | 1.00 (0.85, 1.16) |
| 23.5-26.9 | 91 | | | 66900 | 136.0 | 1.10 (0.89, 1.35) |
| ≥27.0 | 21 | | | 11498 | 182.6 | 1.18 (0.76, 1.81) |
| Total | 328 | | | 315012 | 104.1 | – |
| per 1 SD | – | | | – | – | 1.13 (1.03, 1.25) |
| per 5 kg/m^2^ | – | | | – | – | 1.28 (1.05, 1.55) |
| **Smoking at age 25** | | | |  |  |  |
| <20.0 | | | 72 | 65558 | 109.8 | 0.97 (0.77, 1.23) |
| 20.0-23.4 | | | 20 | 21417 | 93.4 | 1.00 (0.64, 1.56) |
| 23.5-26.9 | | | 47 | 22514 | 208.8 | 1.55 (1.16, 2.07) |
| ≥27.0 | | | 12 | 3722 | 322.4 | 2.38 (1.34, 4.24) |
| Total | | | 151 | 113211 | 133.4 | – |
| per 1 SD | | | – | – | – | 1.29 (1.10, 1.51) |
| per 5 kg/m^2^ | | | – | – | – | 1.63 (1.20, 2.21) |

The models were stratified by age at risk, sex, and region, and adjusted for education, and alcohol. SD for young adulthood adulthood BMI was 2.67 kg/m^2^. *P* for heterogeneity = 0.19.

# Supplementary Table 7. Adjusted HRs for PC by BMI and WC in CKB

|  | **No. events** | **HR (95% CI)** |
| --- | --- | --- |
|  |  |  |
| **BMI at age 25** |  |  |
| <20.0 | 67 | 0.78 (0.61, 0.99) |
| 20.0-22.4 | 175 | 1.00 (0.86, 1.16) |
| 22.5-24.9 | 142 | 1.07 (0.91, 1.26) |
| 25.0-26.9 | 62 | 1.42 (1.10, 1.83) |
| ≥27.0 | 33 | 1.49 (1.05, 2.11) |
| per 1SD | 479 | 1.17 (1.08, 1.28) |
|  |  |  |
| **BMI** |  |  |
| <20.0 | 56 | 0.79 (0.60, 1.03) |
| 20.0-22.4 | 125 | 1.00 (0.84, 1.20) |
| 22.5-24.9 | 128 | 0.91 (0.77, 1.08) |
| 25.0-26.9 | 89 | 1.03 (0.83, 1.26) |
| ≥27.0 | 86 | 0.99 (0.79, 1.23) |
| per 1SD | 484 | 1.07 (0.98, 1.18) |
|  |  |  |
| **Waist circumference** |  |  |
| <70 | 63 | 0.98 (0.74, 1.24) |
| 70-79 | 151 | 1.00 (0.85, 1.18) |
| 80-89 | 161 | 1.09 (0.93, 1.26) |
| 90-99 | 81 | 1.14 (0.91, 1.42) |
| ≥100 | 28 | 1.77 (1.17, 2.52) |
| per 1SD | 484 | 1.07 (0.97, 1.17) |

The models were stratified by age at risk, sex and study area, and adjusted for education, smoking, and alcohol. For young adulthood BMI, smoking status at age 25 was used in the adjustment. SD was 3.38 kg/m^2^ for adulthood BMI, 2.59 kg/m^2^ for young adulthood BMI, and 9.74 cm for WC.

# Supplementary Table 8. Adjusted HRs for PC by tertiles of anthropometric measures at adulthood in CKB

|  | **No. events** | **HR (95% CI)**^1^ |
| --- | --- | --- |
|  |  |  |
| **Hip circumference** |  |  |
| Tertile 1 | 171 | 1.00 (0.84, 1.19) |
| Tertile 2 | 149 | 1.08 (0.93, 1.25) |
| Tertile 3 | 164 | 1.11 (0.92, 1.33) |
| per 1SD | 484 | 1.08 (0.98, 1.19) |
|  |  |  |
| **Waist to hip ratio** |  |  |
| Tertile 1 | 120 | 1.00 (0.83, 1.21) |
| Tertile 2 | 147 | 1.01 (0.86, 1.19) |
| Tertile 3 | 217 | 1.13 (0.98, 1.31) |
| per 1SD | 484 | 1.04 (0.96, 1.14) |
|  |  |  |
| **Body fat percentage** |  |  |
| Tertile 1 | 190 | 1.00 (0.82, 1.22) |
| Tertile 2 | 145 | 1.00 (0.87, 1.14) |
| Tertile 3 | 149 | 1.04 (0.85, 1.28) |
| per 1SD | 484 | 1.03 (0.92, 1.16) |
|  |  |  |
| **Height adjusted weight** |  |  |
| Tertile 1 | 155 | 1.00 (0.82, 1.22) |
| Tertile 2 | 147 | 1.02 (0.89, 1.18) |
| Tertile 3 | 182 | 1.23 (1.02, 1.48) |
| per 1SD | 484 | 1.09 (0.97, 1.23) |
|  |  |  |
| **Height** |  |  |
| Tertile 1 | 194 | 1.00 (0.81, 1.23) |
| Tertile 2 | 178 | 1.07 (0.96, 1.20) |
| Tertile 3 | 213 | 1.28 (1.04, 1.57) |
| per 1SD | 585 | 1.09 (0.96, 1.23) |
|  |  |  |
| **Leg length** |  |  |
| Tertile 1 | 170 | 1.00 (0.83, 1.21) |
| Tertile 2 | 187 | 1.07 (0.95, 1.20) |
| Tertile 3 | 228 | 1.16 (0.97, 1.39) |
| per 1SD | 585 | 1.06 (0.95, 1.18) |

The models were stratified by age at risk, sex and study area, and adjusted for education, smoking, and alcohol. SD was 6.87 cm for HC, 0.07 for WHR, 8.39 for body fat percentage, 10.75 kg for weight, 8.27 cm for standing height, and 4.81 cm for leg length.

# Supplementary Figure 1. PRISMA flow chart of literature search

751 duplicates removed

134 publications were assessed based on full-text and their references were reviewed for additional publications

1478 publications excluded not meeting inclusion criteria:

- Non-human studies: 203
- Did not report on the associations of interest: 782
- Non-original articles: 413
- Not prospective study design: 19
- Non-English articles: 24
- Duplicate: 36
- Cannot be found: 1

**Adulthood BMI:**

29 publications, corresponding to 30 individual studies and 1 pooled analysis, included for adulthood BMI:

- Dose-response meta-analysis: 31 studies

4 4 publications, corresponding to 3 individual studies and 1 pooled analysis, included for young adulthood BMI:

- Dose-response meta-analysis: 4 studies

103 publications excluded:

- Duplicate publications: 27
- Did not provide risk estimates on BMI and PC: 63
- Not prospective study: 13

6 found in bibliographies

**Young adulthood BMI:**

3 publications, corresponding to 2 individual studies and 1 pooled analysis, included for adulthood WC:

- Dose-response meta-analysis: 3 studies

4 3 publications, corresponding to 2 individual studies and 1 pooled analysis, included for adulthood WHR:

- Dose-response meta-analysis: 3 studies

2357 publications identified on initial search:

- 974 Pubmed
- 1383 Embase

1606 abstracts screened based on title and abstract

# Supplementary Figure 2. Mean young adulthood and adulthood BMI by age at baseline in males and females in CKB

**
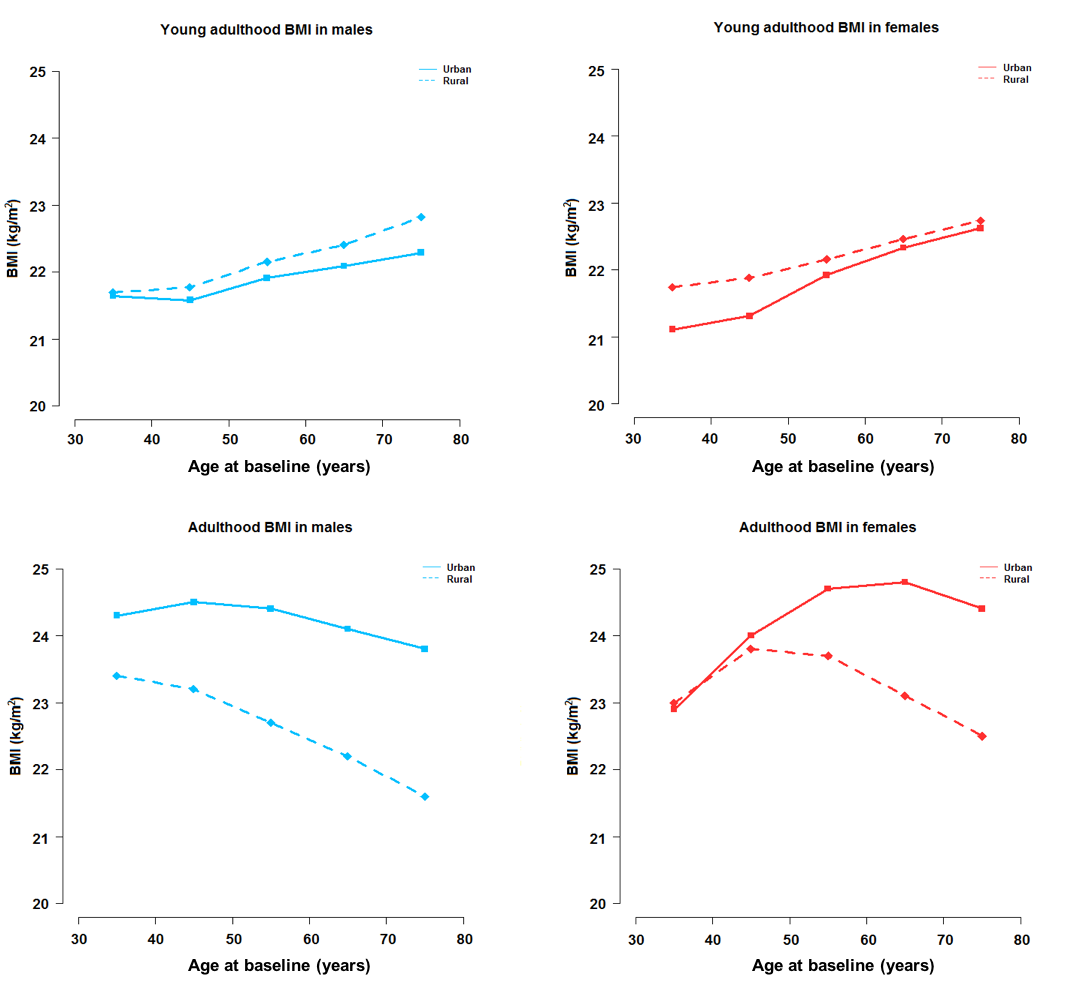
**

# Supplementary Figure 3. Adjusted HRs for PC by tertiles of anthropometric measures at adulthood in CKB

**
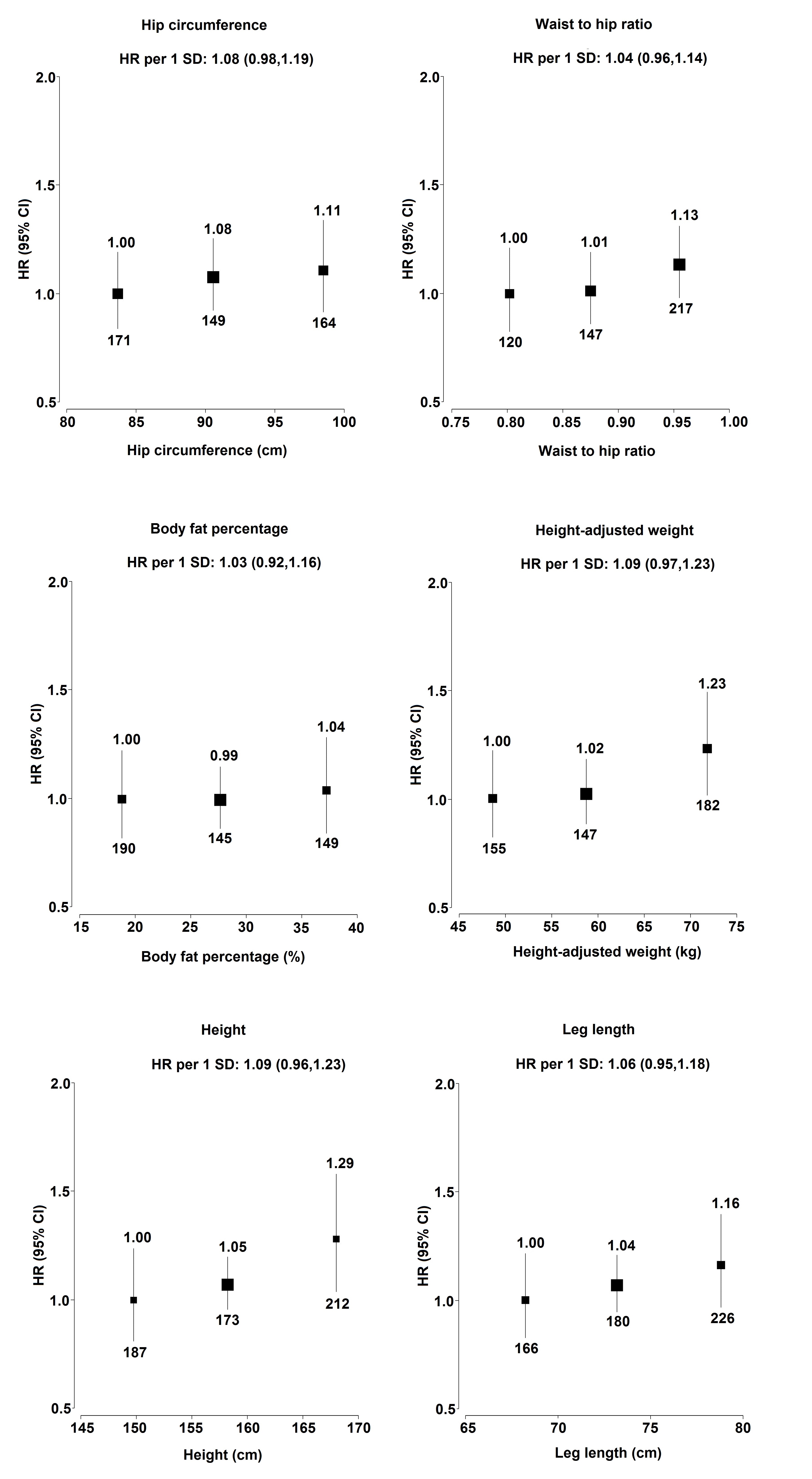
**

SD was 6.87 cm for HC, 0.07 for WHR, 8.39 for body fat percentage, 10.75 kg for weight, 8.27 cm for standing height, and 4.81 cm for leg length. The HRs and CIs are shown in Supplementary Table 7.

# Supplementary Figure 4. Funnel plot of published studies of young adulthood BMI and PC


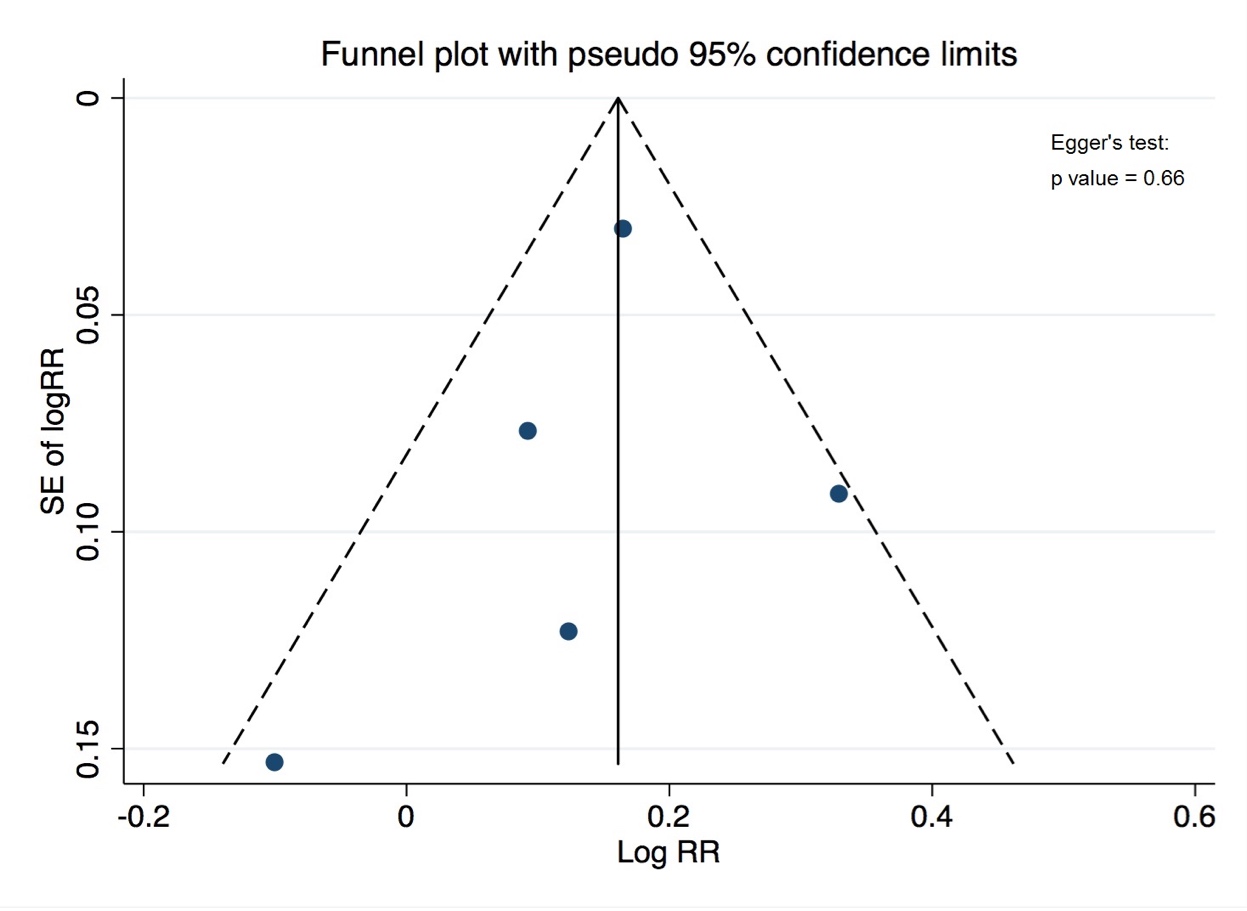


# Supplementary Figure 5. Adjusted RRs for PC associated with a 5 units higher adulthood BMI, measured or self-reported, in meta-analysis of CKB and 31 published studies

**
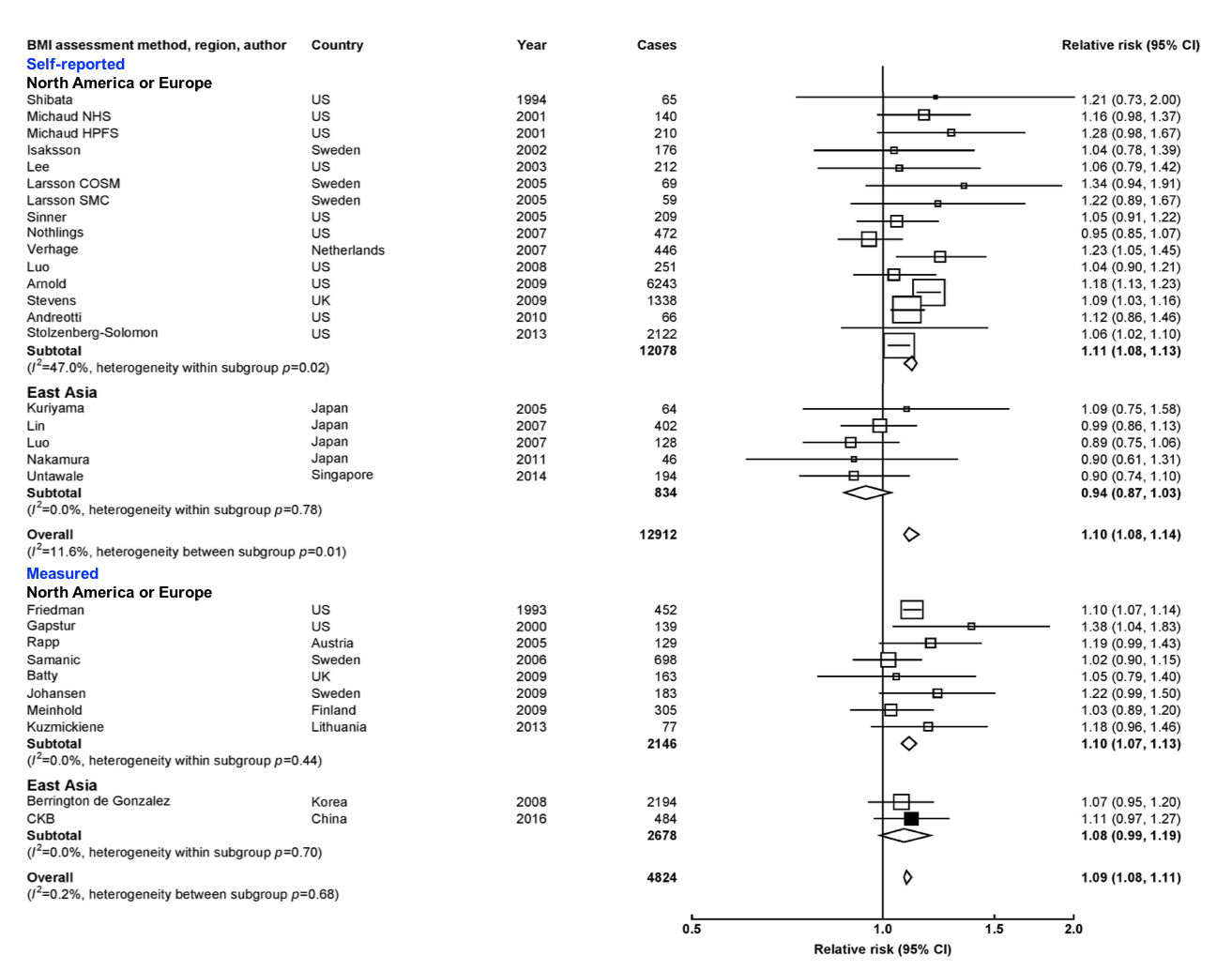
**

# Supplementary Figure 6. Funnel plot of published studies of adulthood BMI and PC

1. Asia

**
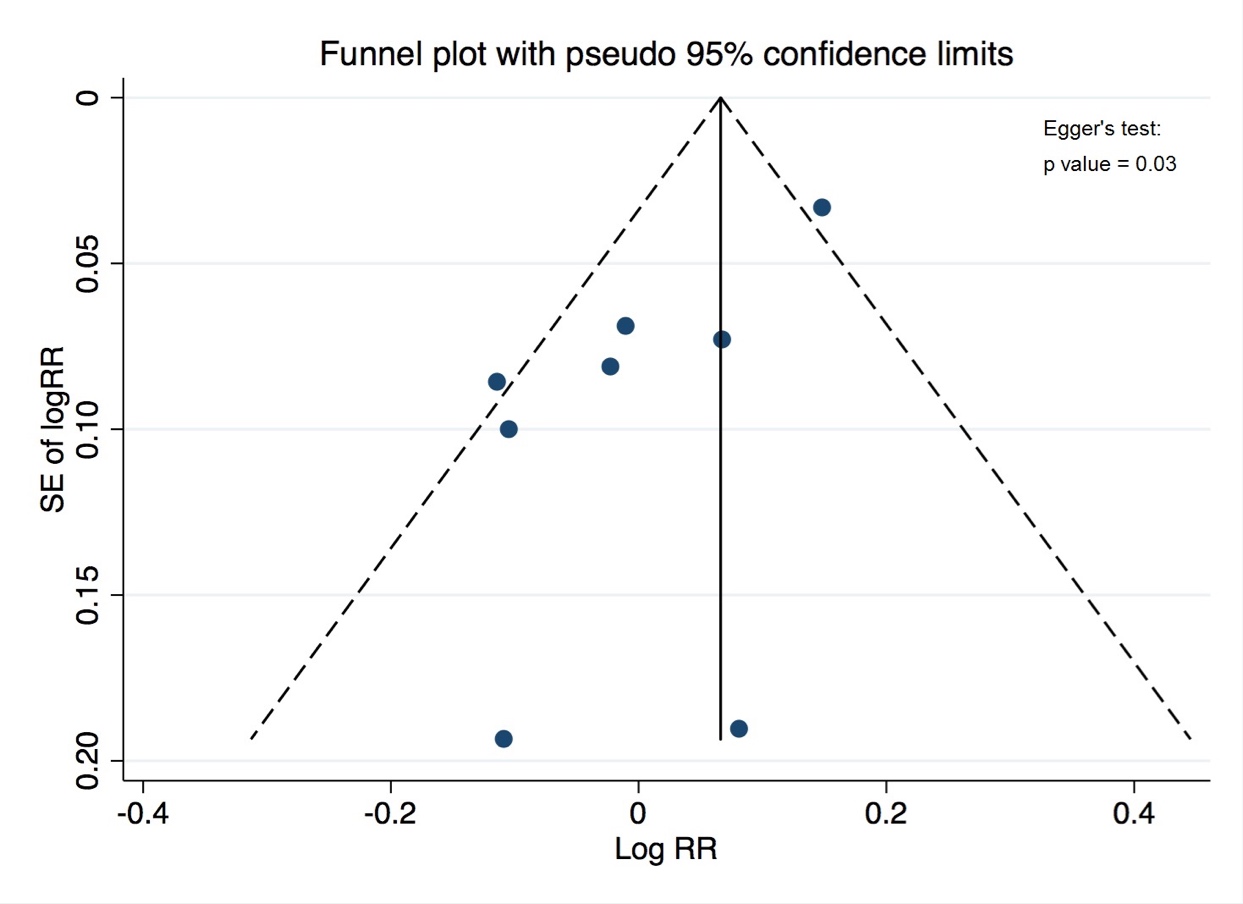
**

1. North America or Europe

**
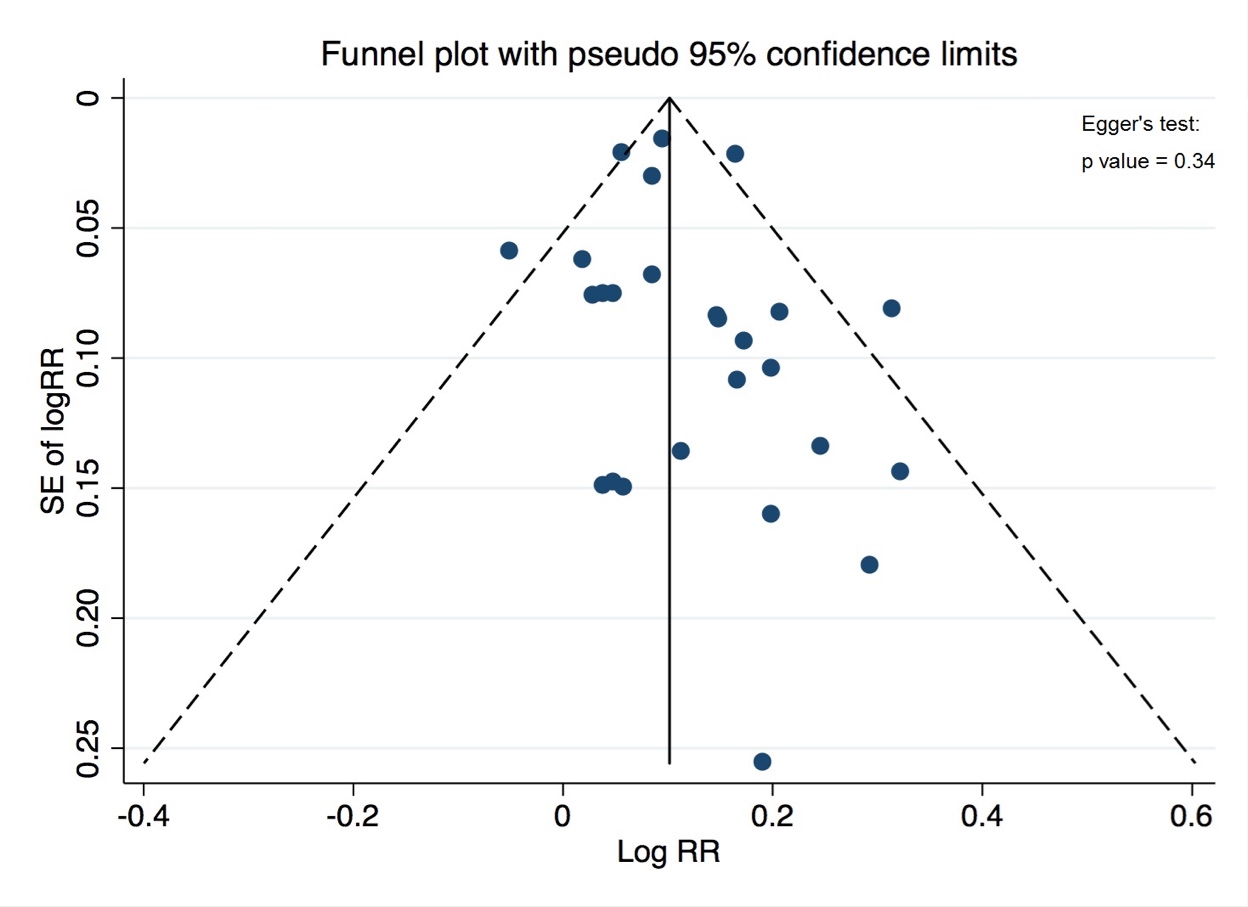
**

# Supplementary Figure 7. Adjusted RRs for PC associated with 1 SD higher adulthood BMI, measured or self-reported, in meta-analysis of CKB and 31 published studies

**
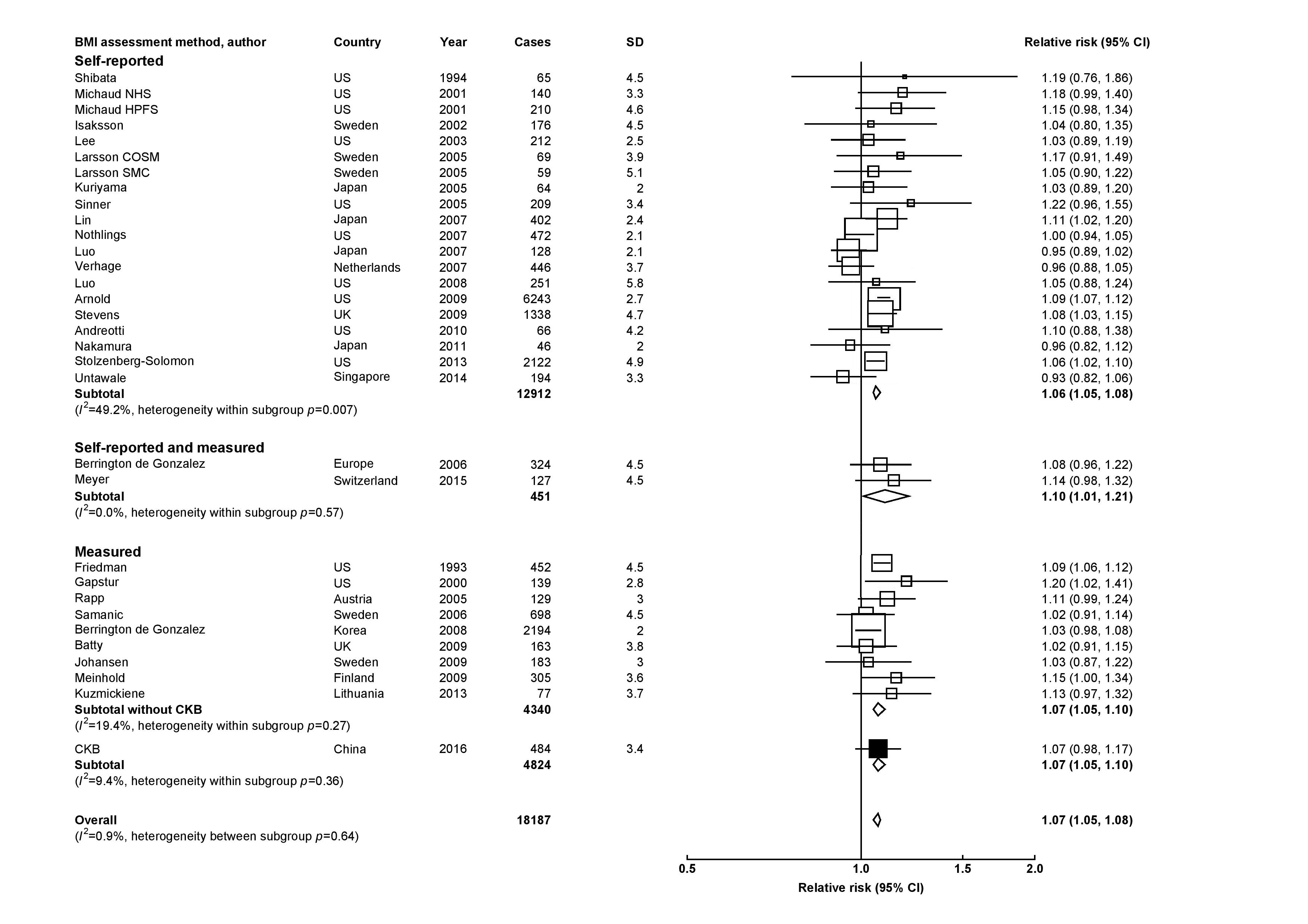
**

# Supplementary Figure 8. Adjusted RRs for PC associated with a 5 units higher adulthood BMI in meta-analysis of CKB and 27 published studies, by study-level mean BMI

**
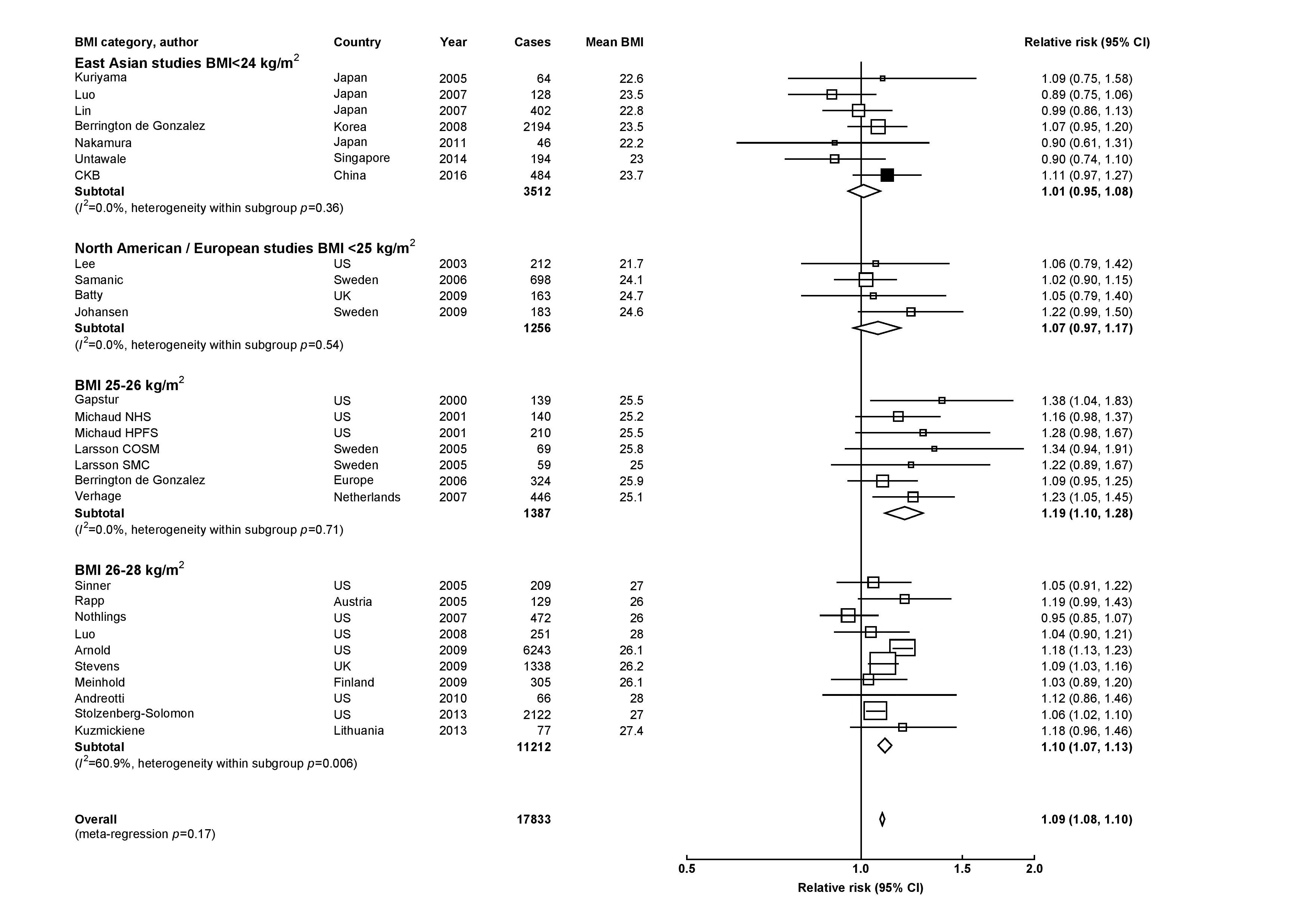
**

* Mean BMI was not reported in four studies.

# Supplementary Figure 9. Adjusted RRs for PC associated with a 5 units higher adulthood BMI in meta-analysis of CKB and 31 published studies, by median follow-up (years)


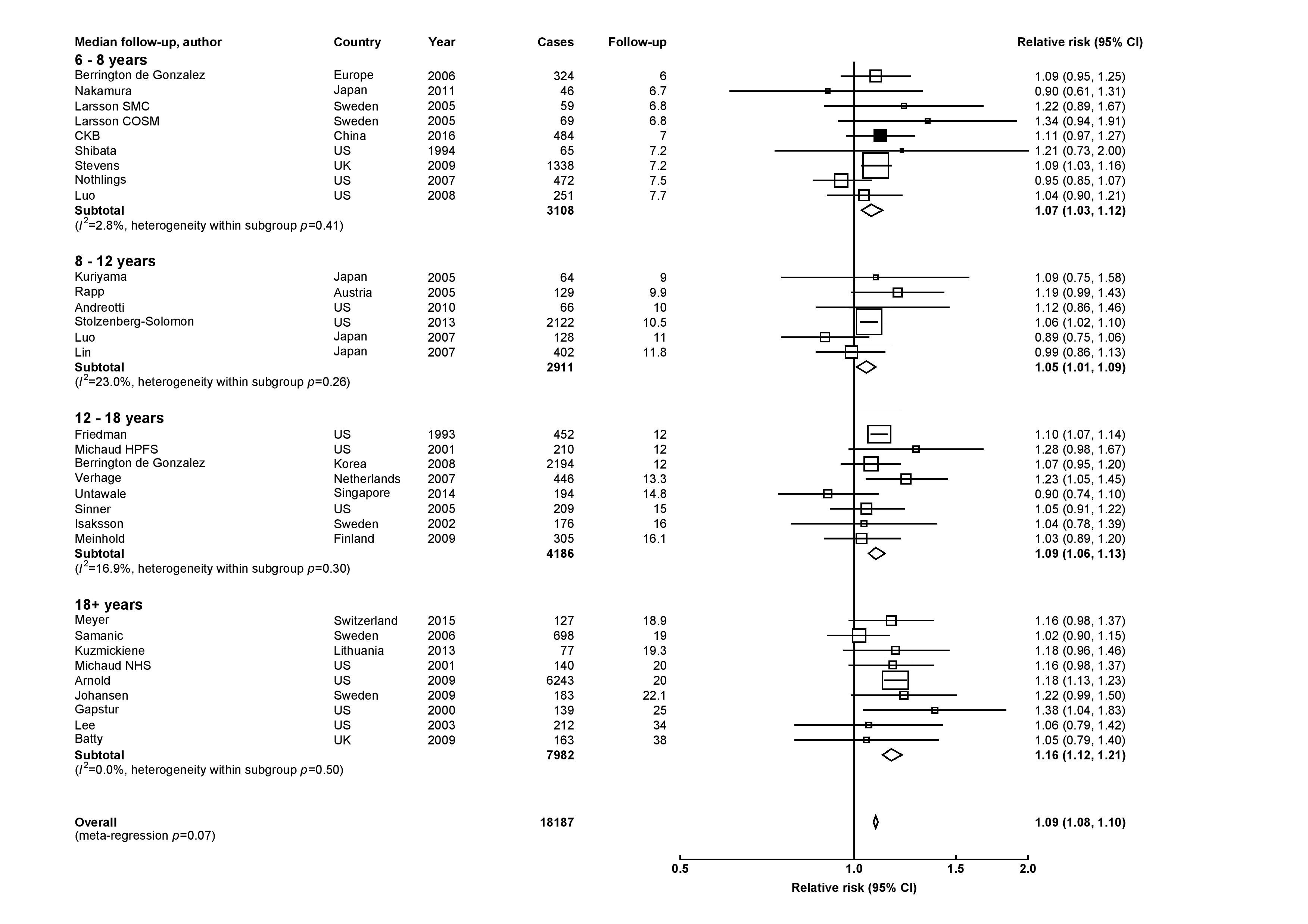


# Supplementary Figure 10. Adjusted RRs for PC associated with a 5 units higher adulthood BMI in meta-analysis of CKB and 31 published studies, by mean age of each study

**
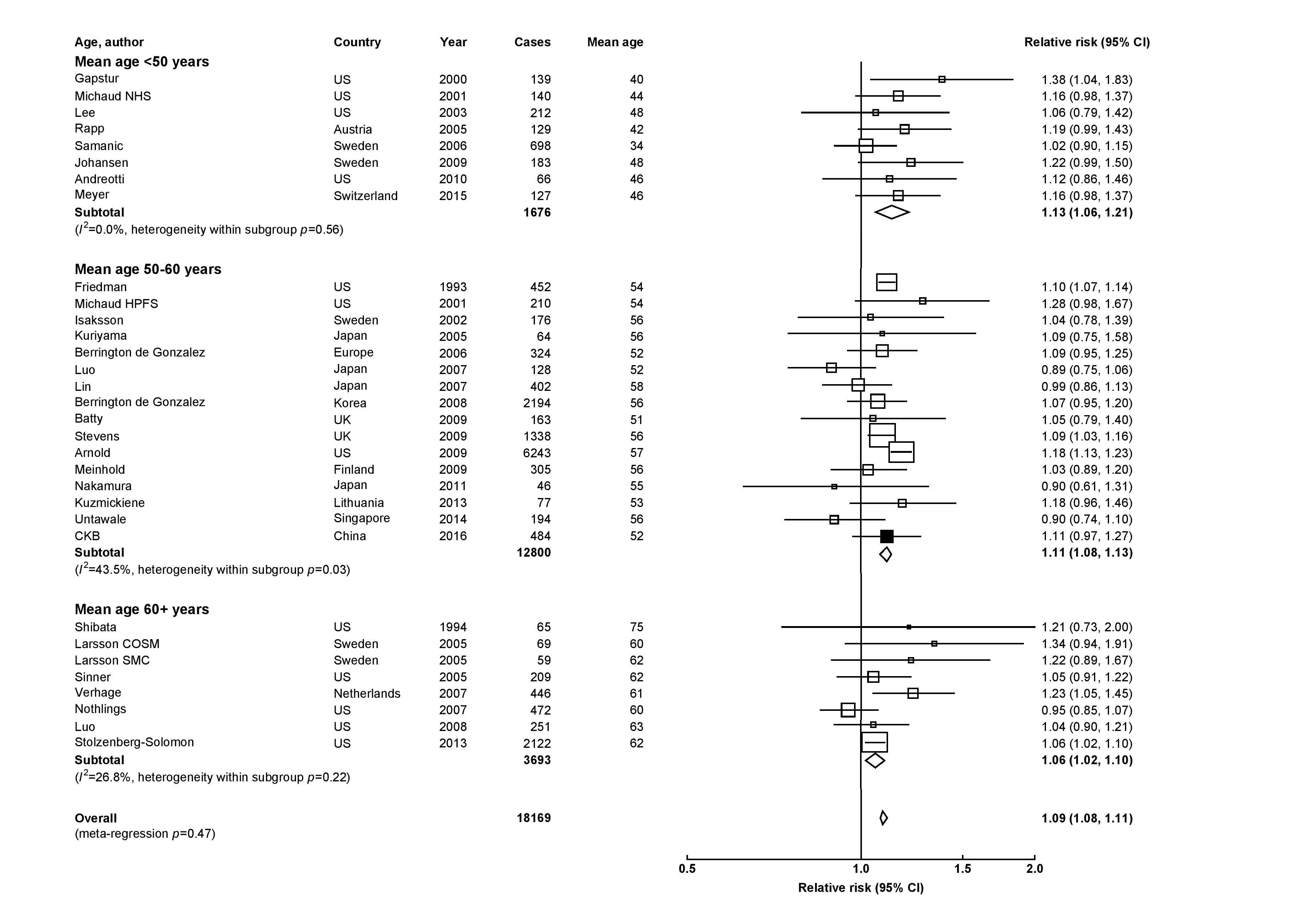
**

# Supplementary Figure 11. Adjusted RRs for PC associated with a 10 cm higher WC and a 0.1 higher WHR in meta-analysis of CKB and 3 published studies, with or without adjustment for BMI


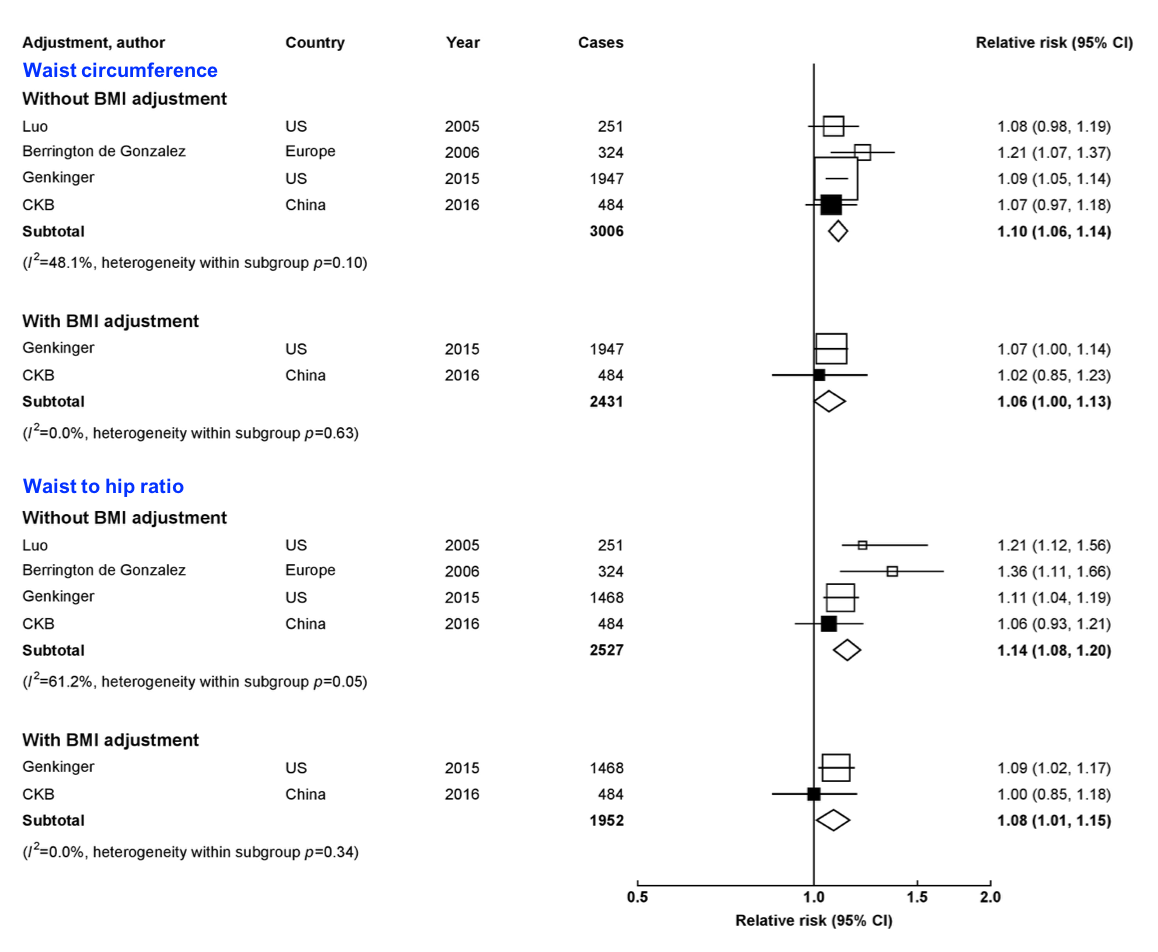

Supplement: Supplementary file 1 [file jech-2017-208895supp001.docx]
